# Genetic dissection of QTLs and differentiation analysis of alleles for heading date genes in rice

**DOI:** 10.1371/journal.pone.0190491

**Published:** 2018-01-03

**Authors:** Hua Zhang, Shuyi Liu, Gao Chen, Xu Liu, Ning Xuan, Yongyi Yang, Wei Liu, Hanfeng Ding, Fangyin Yao

**Affiliations:** 1 Bio-technology Research Center, Shandong Academy of Agricultural Sciences, Jinan, Shandong, P. R. China; 2 Shandong Center of Crop Germplasm Resources, Shandong Academy of Agricultural Sciences, Jinan, Shandong, P. R. China; National Cheng Kung University, TAIWAN

## Abstract

Heading date is an important agronomic trait in rice (*Oryza sativa* L.); it determines the geographical and seasonal adaptability of the crop. Single segment substitution lines (SSSLs) have become the preferred experimental materials in mapping functional genetic variations as the particular chromosome segments from donor genotypes can be evaluated for their impact on the phenotype in a recurrent recipient background. The phenotypic differences can be attributed to the control of quantitative trait loci (QTLs). Here, we evaluated a library consisting of 1,123 SSSLs in the same genetic background of an elite rice variety, Huajingxian74 (HJX74), and revealed four SSSLs, W05-1-11-2-7-6 (W05), W08-16-3-2 (W08), W12-28-58-03-19-1 (W12), and W22-9-5-2-4-9-3 (W22), which had a significantly different heading date compared to HJX74. To further genetically dissect the QTLs controlling heading date on chromosomes 3, 6, and 10, four SSSLs were used to develop 15 secondary SSSLs with the smaller substituted segments. The *qHD-3* heading date QTL detected in W05 and W08 was delimited to an interval of 4.15 cM, whereas *qHD-6-1* and *qHD-6-2* heading date QTLs dissected from the substituted segments in W12 were mapped to the intervals of 2.25-cM and 2.55-cM, respectively. The *qHD-10* QTL detected on the substituted segment in W22 was mapped to an interval of 6.85-cM. The nucleotide and amino acid sequence changes for those genes in the secondary SSSLs were also revealed. The allele variations of those genes might contribute to the heading date QTLs on chromosome 3 (*DTH3*, *OsDof12*, and *EHD4*), chromosome 6 (*Hd3a*, *Hd17*, and *RFT1*), and chromosome 10 (*Ehd1* and *Ehd2*). These sequence variations in heading date genes would be useful resources for further studying the function of genes, and would be important for rice breeding. Overall, our results indicate that secondary SSSLs were powerful tools for genetic dissection of QTLs and identification of differentiation in the genes.

## Introduction

Rice (*Oryza sativa* L.) is a cereal crop used as a staple food for more than half of the population in the world, and is therefore important for global food security [1, 2]. Heading date is a valuable agronomic trait, and it determines the geographic adaptability of rice although it is influenced by regional and seasonal factors. Several genes and genetic loci that regulate heading date have been identified in past decades using molecular genetics [3–8].

Many important traits in rice, such as yield, heading date, grain quality, and culm length, are regulated by multiple genes, representing quantitative traits with complex inheritances. It is difficult to identify these genes because their individual effects on these phenotypes are relatively small. QTLs with major effects can be detected in the primary populations such as F_2_ and F_3_ mapping hybrids, doubled haploid lines, and recombinant inbred lines; however, QTLs with minor effects or epistatic interactions might not be detected in these materials [9, 10]. The applications of secondary mapping populations, such as the introgression lines in tomato (*Solanum lycopersicum*) [11] and oilseed rape (*Brassica napus*) [12], the chromosome substitution lines in *Arabidopsis thaliana* [13], and the chromosome segment substitution lines in rice, have shown the distinct advantages for QTL analysis [14,15]. Secondary segregation materials like F_2_ and F_3_ populations can be developed from a cross between a near isogenic line (NIL) with the target QTL and the recurrent parent, which can be used to identify recombinants with the introgression segment using flanking markers [16]. In the present study, SSSLs were developed through successive backcrossing of the recipient parent with the donor genotype followed by marker-assisted selection. Each SSSL contained homozygous genetic background from its recipient except the substituted segment from a donor, and could be used to dissect polygenic traits into a set of monogenic loci. Furthermore, the epistatic effects from the donor parent were eliminated. Therefore, SSSLs developed recently are powerful tools for analyzing genetic mechanisms of QTLs and elucidating gene functions in plants [17–19].

The secondary mapping populations comprise single segment substitution lines, of which each contains a single homozygous marker-defined chromosome segment from a donor parent in a recurrent parent background. These populations can be used to further dissect QTLs for complex traits. Recently, a library of 1,123 SSSLs have been constructed in rice using the elite *indica* variety Huajingxian74 (HJX74) as a recipient, and 24 varieties (14 *indica* and 10 *japonica*) were collected from around the world as donors of chromosome segments. The size of the substituted segments in the SSSLs was between 0.15 cM and 109.7 cM, with an average size of 19.3 cM. The total length of the substituted segments in the SSSL library was 21,674 cM as equate to approximately 14 times the size of the rice genome [20]. The high level of uniformity in the SSSL genetic background other than the single substituted segment supported that all the phenotypic variations were associated with the substituted segments from the donors [11, 21]. This SSSL population has therefore been widely used to map QTLs [22].

It is difficult to determine whether a QTL consists of a single gene or a cluster of genes with relatively weak effects; for example, *Hd3* on chromosome 6 in rice was originally considered to be a single gene controlling heading date, but genetic dissection revealed that it contained two tightly linked genes, *Hd3a* and *Hd3b* [23]. In this study, four rice SSSLs had significantly different heading dates from the recurrent parent HJX74, indicating the presence of heading date QTLs in the substituted regions. Then 15 secondary SSSLs were developed with smaller substitution segments from these four primary SSSLs, and used to further dissect these heading date QTLs in rice. To elucidate the genetic basis of the rice heading date, eight rice heading date genes were cloned from HJX74 and the corresponding SSSLs including *DTH3* (*Days to heading on chromosome 3*), *OsDof12* (*DNA-binding with one finger 12*), *Ehd4* (*Early heading date 4*), *Hd3a* (*Heading date 3a*), *Hd17*, *RFT1*, *Ehd1* and *Ehd2*, and a comprehensivecomparison of the nucleotide sequences of these genes between genotypes was performed. Furthermore, multiple features of the proteins encoded by these genes were investigated, which included amino acid composition and the physicochemical, hydrophobic and hydrophilic properties, and the secondary structure. In addition, the functions of these genes in regulation of heading date were also discussed.

## Materials and methods

### Plant materials

The library of rice SSSLs was constructed by our group [17, 20], and four lines with different heading dates to the recurrent parent HJX74, W05-1-11-2-7-6 (W05), W08-16-3-2 (W08), W12-28-58-03-19-1 (W12), and W22-9-5-2-4-9-3 (W22) [17], were selected for use in the development of secondary SSSLs. The four SSSLs were backcrossed to HJX74, and four F_2_ and F_3_ segregating populations were used to identify secondary SSSLs through simple sequence repeat (SSR) marker-assisted selection. A total of 15 secondary SSSLs were developed from those four crosses. A total of 150 F_2_ plants from each cross were grown in field conditions at Yinma Spring, Shandong Academy of Agriculture Science, Jinan, P. R. China (latitude: 36.40° N, longitude: 117° E), with a day length of over 14 h. For all genotypes, 60 plants were planted 16.6 cm apart in six rows, with 25 cm between rows. Data were collected from the 20 plants in the middle of each plot.

### Molecular marker assay

DNA was extracted from fresh leaves harvested from each plant using the micro-isolation method described by Zheng et al. [24], with minor modification. PCR amplification was conducted following the procedure of Panaud et al. [25], with little modification. After separating the amplified products by electrophoresis, the gels were subjected to the silver staining process described by Li et al. [26]. SSR markers were used to determine the genotypes of the secondary SSSLs at these marker loci. The required density of molecular markers was achieved using previously published SSRs [27] in combination with the SSR and InDel markers developed at the Plant Molecular Breeding Research Center, South China Agricultural University, Guangzhou, China [28].

### Detection of QTLs

QTLs were detected by quantifying the number of days to heading in each line and using a Dunnett’s test to reveal statistically significant differences between each SSSL and the control, HJX74. A probability level of *P* ≤ 0.01 was used as the threshold for presence of putative QTLs, which would be located in the substituted segment of the SSSLs. Substitution mapping for QTLs was carried out following the methods described by Wissuwa et al. [29]. If one QTL could be detected in multiple SSSLs containing overlapping substituted segments, the QTL was mapped to the interval of overlap. If a QTL was detected in only one SSSL and was not detected in other overlapping SSSLs, the QTL was mapped to the non-overlapping region of the substituted segments in the QTL-containing SSSL. The additive effect and additive contribution (%) of a QTL was estimated following the method described by Eshed and Zamir [30]:

$$\begin{array}{l} \mathrm{Additive}\;\mathrm{effect}\;\mathrm{on}\;\mathrm{heading}\;\mathrm{date}\;(\mathrm{days})=(\mathrm{days}\;\mathrm{to}\;\mathrm{heading}\;\mathrm{of}\;\mathrm{an}\;\mathrm{SSSL}\;\text{-}\;\mathrm{days}\;\mathrm{to}\;\mathrm{heading}\;\mathrm{of}\;\mathrm{the}\;\mathrm{recipient})/2.\\ \mathrm{Additive}\;\mathrm{contribution}\;\left(\%\right)=\left(\mathrm{additive}\;\mathrm{effect}/\mathrm{heading}\;\mathrm{dates}\;\mathrm{of}\;\mathrm{the}\;\mathrm{recipient}\right)\times 100. \end{array}$$

QTL nomenclature was conducted using the method described by McCouch et al. [27].

### Primer design

The sequences of the heading date genes (*DTH3*, *OsDof12*, *Ehd4*, *Hd3a*, *Hd17*, *Ehd1*, *RFT1*, and *Ehd2*) were downloaded from a publicly available rice genome sequence (http://www.ncbi.nlm.nih.gov). All primers were designed using Primer Premier version 5.0 (Premier Biosoft, Palo Alto, CA, USA) in accordance with the following parameters: 18–25 nucleotides in length, devoid of secondary structure, a GC content of around 50%, and a melting temperature of around 55°C. The primers used for PCR were listed in S1 Table.

### RNA isolation, cDNA synthesis, and PCR amplification

Total RNA was extracted using Trizol reagent (ThermoFisher Scientific, Waltham, MA, USA), following the manufacturer’s instructions. First strand cDNA was synthesized using M-MLV reverse transcriptase and modified oligo (dT), following the manufacturer’s instructions (TaKaRa Biotechnology, Dalian, China). Fragments of the rice heading date genes were amplified using the following PCR program: an initial denaturation cycle at 94°C for 5 min, followed by 35 cycles of 94°C for 30 s, 60°C for 35 s, and 72°C for 1 min, and then a final extension at 72°C for 10 min. The amplified products were separated on 1.0% agarose gel and target bands were purified using E.Z.N.A.® Gel Extraction Kit (Omega Bio-tek’s, Norcross, USA). Then the DNA fragments were cloned into a pGEM-T Easy Cloning Vector (Promega, Madison, WI, USA), transformed into *Escherichia coli* DH5α (TransGen Biotech Co., Ltd., Beijing, China), and sequenced following the techniques outlined by the Bio-Tech Research Center, Shandong Academy of Agricultural Science, Jinan, China.

### Sequence alignment and physicochemical properties

DNAman software (Lynnon Biosoft, San Ramon, CA, USA) was used to analyze the rice heading date gene sequences. The multi-sequence alignment was conducted using Clustal X [31] and the results of alignment were displayed by GeneDoc [32]. Gene structure was displayed using GSDS 2.0 online (http://gsds.cbi.pku.edu.cn/) [33].The analysis of the physicochemical properties was performed using ProtParam (http://web.expasy.org/cgi-bin/protparam/protparam), while the hydrophobic and hydrophilic analyses were conducted using ProtScale (http://web.expasy.org/cgi-bin/protscale/protscale.pl). Porter (http://distill.ucd.ie/porter/) was used to predict protein secondary structure.

## Results

### Identification of QTLs for heading date based on SSSLs

Heading date in the library of rice SSSLs was scored by our previous study [17]. Among the SSSLs, four SSSLs, W05, W08, W12, and W22, showed a significant difference in their heading dates from that observed for the recipient parent variety HJX74 (Table 1). W05 contained a substituted segment on chromosome 3 from its donor variety Lemont., The segment had an estimated length of 52.15 cM. It took 121 days for W05 to reach the heading stage, which was significantly longer than that of HJX74. W08 had an earlier heading date than HJX74, taking just 92 days; it contained a 22.50-cM substituted segment of chromosome 3 from the donor variety, IR64. W12 took 123 days before heading, and carried a substituted segment from the donor, IR58025B, on chromosome 6, with an estimated length of 10.35 cM. The heading time for W22 was 113 days; this SSSL has an estimated 16.25-cM long substituted segment from its donor, Khazar, on chromosome 10. These results indicated that at least one QTL for heading date was located on the substituted segments in each of the four SSSLs.

**Table 1:** The substituted segments in the four SSSLs and their days to heading in 2014 and 2015.

SSSL	Donor	Chr	Substituted segment Marker tested	Estimated length (cM)	Days to heading (*P*-value)	
					2014	2015
W05	Zihui 100	3	Short arm-RM569-PSM429 -RM232-RM563—RM282	52.15	121.20±0.39 (0.00)	123.20±0.62 (0.00)
W08	IR64	3	Short arm—PSM306-RM569 -RM489—RM545	22.50	92.36±0.86 (0.00)	92.28±0.56 (0.00)
W12	IR58025B	6	RM170—RM190-RM217—RM314	10.35	123.49±0.95 (0.00)	124.49±0.45 (0.00)
W22	Khazar	10	RM596—RM271-RM269-RM258 -RM304—PSM167	16.25	113.42±0.81 (0.00)	112.69±0.76 (0.00)
HJX74	-	-	-	-	105.18±0.59	106.65±0.75

### Identification of QTLs for heading date based on secondary SSSLs

**Fig 1:**
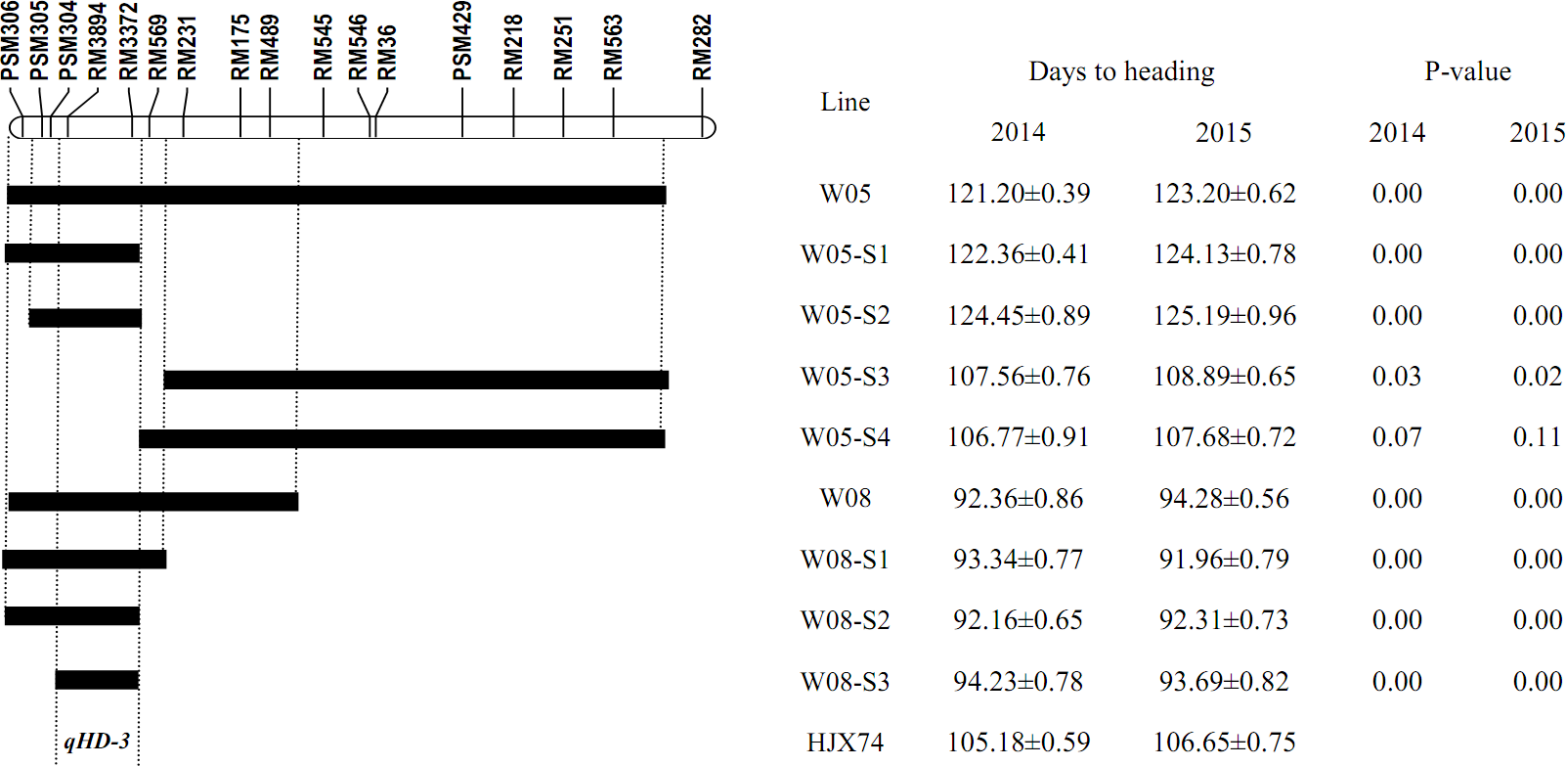
Genetic dissection and fine mapping of QTLs for heading date on rice chromosome 3.

**Fig 2:**
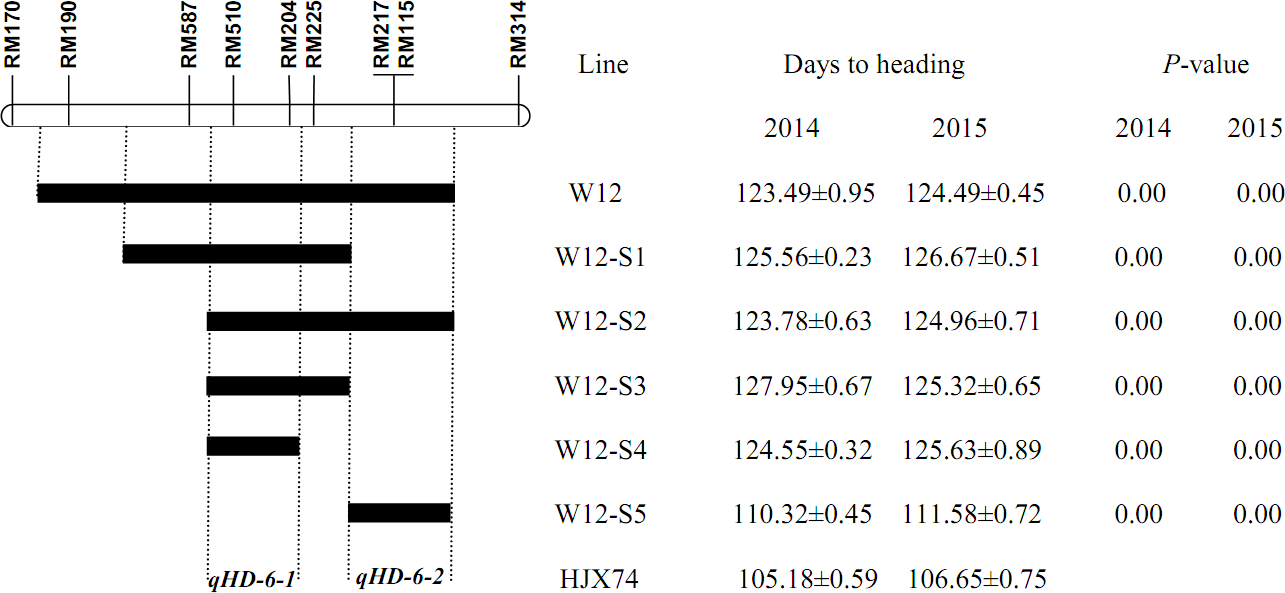
Genetic dissection and fine mapping of QTLs for heading date on rice chromosome 6.

**Fig 3:**
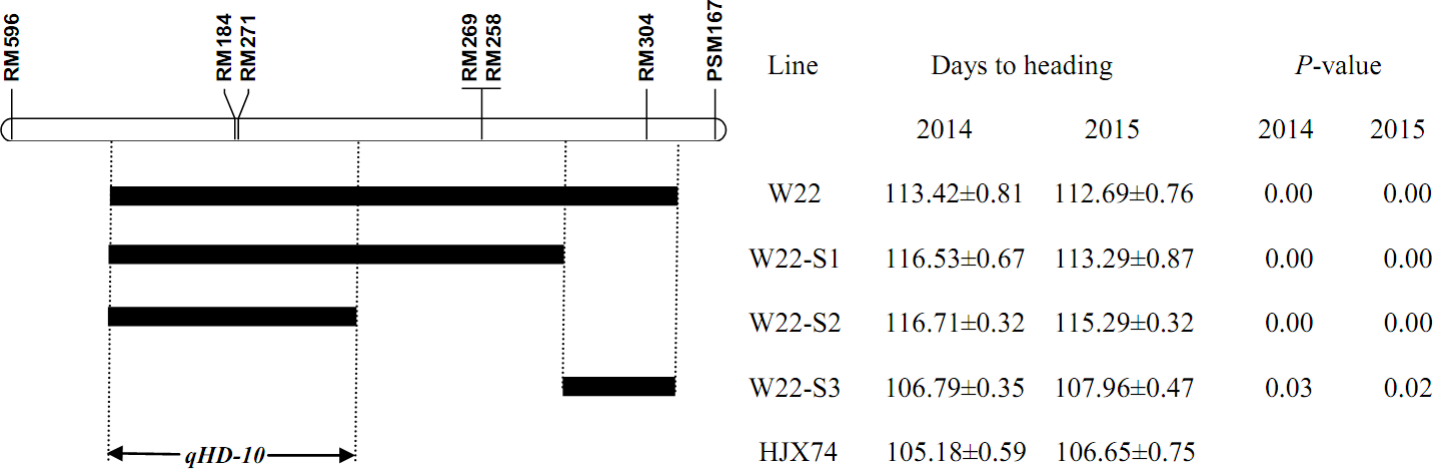
Genetic dissection and fine mapping of QTLs for heading date on rice chromosome 10.

Fifteen secondary SSSLs were developed from the four primary SSSLs (Figs 1–3). The primary SSSL W05 was used to generate four secondary SSSLs with an average substituted segment length of 25.10 cM. Three secondary SSSLs were derived from the primary SSSL W08, which contained substituted segments with an average length of 6.73 cM, whereas the five secondary SSSLs derived from W12 contained an average of 4.01 cM of the substituted segments. Three secondary SSSLs with an average length of the substituted segment of 7.70 cM were developed from the primary SSSL W22. Compared with the length of substituted segments in the primary SSSLs, those of the secondary SSSLs were much shorter; the average length of the substituted segments in the secondary SSSLs was 11.16 cM (range: 4.01 cM to 25.10 cM), whereas the average length of the substituted segments in the primary SSSLs was 25.31 cM (range: 10.35 cM to 52.15 cM; Table 1).

### Genetic dissection and fine mapping of QTLs for heading date using secondary SSSLs

Secondary SSSLs of the substituted segments of chromosome 3 in W05 and W08 were used to identify QTLs for heading date. Lines W05-S1 and W05-S2 showed later heading than the recipient HJX74, whereas those of W05-S3 and W05-S4 were similar to that of HJX74, suggesting the presence of a heading date QTL on the substituted interval of PSM306—PSM305-RM3372—RM569 (double hyphen represents a marker located outside the substitution segment and the single hyphen represents markers known to be associated with the substitution segments) (Fig 1). The secondary SSSLs W08-S1, W08-S2, and W08-S3 had earlier heading dates than HJX74, indicating a heading date QTL on the substituted interval of PSM304—RM3894-RM3372—RM569 (Fig 1). Taken together, these results demonstrated that there was a heading date QTL located in the 4.15-cM overlapping interval of chromosome 3 substituted segments in W05 and W08, PSM304—RM3894-RM3372—RM569, and the QTL was referred to as *qHD-3* hereafter. The allele at the *qHD-3* locus from IR64 promoted earlier heading, whereas the sequence of *qHD-3* derived from Lemont showed that it was responsible for the delayed heading (Fig 1 and Table 2).

**Table 2:** Mapping intervals and allelic effects of QTLs for heading date in the SSSLs.

Line	QTL	Interval mapped (cM)	Allelic effect					
			Days to heading		Additive effect(days)		Additive contribution (%)	
			2014	2015	2014	2015	2014	2015
W05-1-11-2-7-6	*qHD-3*	4.15	121.20±0.39	123.20±0.62	8.01	8.28	7.62	7.76
W08-16-3-2	*qHD-3*	4.15	92.36±0.86	94.28±0.56	-6.41	-6.18	-6.09	-6.72
W12-28-58-03-19-1	*qHD-6-1*	2.25	123.49±0.95	124.49±0.45	9.15	8.92	8.7	8.36
W12-28-58-03-19-1-S5	*qHD-6-2*	2.55	110.32±0.45	111.58±0.72	2.57	2.47	2.44	2.32
W22-9-5-2-4-9-3	*qHD-10*	6.85	113.42±0.81	112.69±0.76	4.12	3.02	3.92	3.68
HJX74			105.18±0.59	106.65±0.75				

To identify the QTL for heading date on the substituted segment of chromosome 6 in W12, five secondary SSSLs were surveyed. Among them, W12-S1, W12-S2, W12-S3, and W12-S4 had significantly different heading dates compared to HJX74 (Fig 2). Their substituted segments were shorter than those of W12, and overlapped at the interval of RM587—RM510-RM204—RM225, indicating a QTL for heading date in this 2.25-cM segment of chromosome 6 (*qHD-6-*1). Furthermore, another secondary SSSL, W12-S5, had an earlier heading date than W12 but later than HJX74. This line contained a short substituted segment of RM225—RM217-RM115—RM314 on chromosome 6, which did not overlap in *qHD-6-1*, then a second minor QTL for heading date at this 2.55-cM interval was referred to as *qHD-6-2*. The additive effect and additive contribution of the major QTL, *qHD-6-1*, to heading date,were 9.15 days and 8.70%, respectively, whereas the minor QTL, *qHD-6-2*, had an additive effect of 2.57 days and an additive contribution of 2.44%. For both loci, the alleles from IR58025B resulted in delayed heading. The *qHD-6-1*-containing SSSLs, W12-S1, W12-S2, W12-S3, and W12-S4 had similar heading dates to W12, whereas the *qHD-6-2*-containing SSSL, W12-S5, showed earlier heading date than W12, demonstrating that there was an epistatic effect of *qHD-6-1* over *qHD-6-2* (Fig 2 and Table 2).

To identify the QTL for heading date on the substituted segment of chromosome 10 in W22, three secondary SSSLs were analyzed. W22-S1 and W22-S2 showed a significant difference in heading date and *P*-value (<0.01) compared to HJX74 (Fig 3). Their substituted segments overlapped at the interval of RM596—RM184-RM271—RM269. The third SSSL, W22-S3, contained a substituted segment at RM269—RM304—PSM167, but had a similar heading date to HJX74. This region did not contain a QTL for heading date. The *qHD-10* QTL on the substituted segment in W22 was therefore located in the 6.85-cM interval of RM596—RM184-RM271—RM269 (Fig 3 and Table 2).

### Differentiation of *DTH3*, *OsDof12* and *EHD4* in the SSSLs and the recurrent parent

Because secondary SSSLs had the smallest substitution fragments, these particular secondary SSSLs were used to clone the heading date genes. *DTH3*, *OsDof12* and *EHD4* had been previously mapped to the QTL region flanked by PSM306 and RM569. We used secondary SSSL W05-S2 to clone the heading date genes (Table 3).

**Table 3:** Putative candidate genes of each QTL.

QTL	Line used for gene clone	Causal gene	Nucleotide variation	amino acid (aa) variation
*qHD-3*	W05-S2	*DTH3* [38]	missing of 627 nucleotides in W05-S2	little similarity between HJX74 and W05-S2
		*OsDof12* [39]	seven SNP	three aa substitutions
		*Ehd4* [4]	3-bp deletion in HJX74, 3-bp deletion in W05-S2 and 13 SNPs	an aa omitted in HJX74, an aa omitted in W05-S2 and six aa substitutions
*qHD-3*	W08-S3	*DTH3* [38]	missing of 612 nucleotides in W08-S3	little similarity between HJX74 and W08-S3
		*OsDof12* [39]	seven SNP	four aa substitutions
		*Ehd4* [4]	3-bp deletion in HJX74, 3-bp deletion in W08-S3 and 10 SNPs	an aa omitted in HJX74, an aa omitted in W08-S3 and five aa substitutions
*qHD-6-1*	W12-S4	*Hd3a* [8,23]	four fragments missing in HJX74 and three nucleotides substitution	Coding terminated prematurely
		*Hd17* [3]	four fragments missing in HJX74 and five SNP variations	Coding terminated prematurely
		*RFT1* [40]	12 SNP variations	five amino acid mutation
*qHD-10*	W22-S2	*Ehd1* [7]	completely consistent	completely consistent
		*Ehd2* [41]	229 nucleotides long deletion in W22-S2 and three SNPs	frameshift

**Fig 4:**
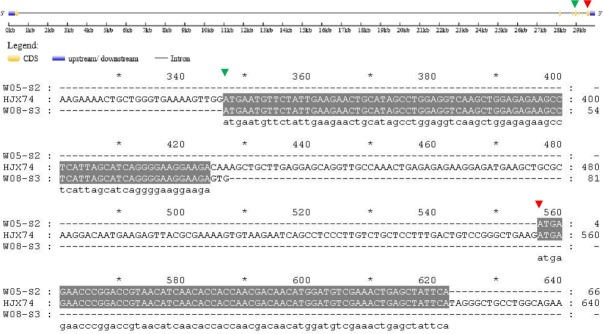
Alignment of *DTH3* coding sequences from HJX74, W05-S2 and W08-S3. The green triangles indicate the locus of CDS sequence from W08-S3, whereas red triangles indicate the locus of CDS sequence from W05-S2.

In HJX74, the full length of the rice heading date gene *DTH3* was 693 bp; however, in the secondary SSSL W05-S2, *DTH3* was only 66 bp, showing a large deletion of 627 nucleotides. The W05-S2 sequence missed nucleotides 1–556 and 623–693, but the remaining 66 bp was identical to the sequence of HJX74 (Fig 4, Table 3).

**Fig 5:**
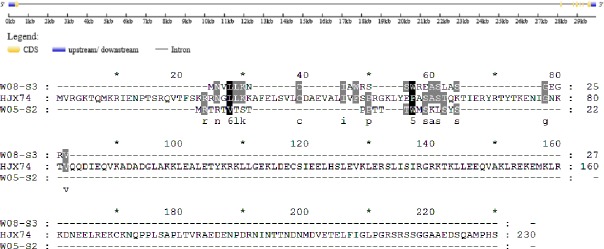
Alignment of DTH3 amino acid sequences from HJX74, W05-S2 and W08-S3.

The amino acid sequences of DTH3 in W05-S2 and HJX74 had major differences; in HJX74, the translated DTH3 protein comprised 230 amino acids, whereas the W05-S2 sequence encodes just 22 amino acids (Fig 5, Table 3). The shifted reading frame indicated that few of the amino acids in the two genotypes were the same even in this shorter sequence, therefore, it was difficult to compare the conformation and structure of the two DTH3 proteins, which were likely affecting the heading date of these rice genotypes.

In the secondary SSSL W08-S3, the full length of *DTH3* was 81 bp, which was 612 nucleotides shorter than the sequence of *DTH3* from HJX74. In HJX74, the 78 nucleotides between nucleotides 347 to 424 were identical to those in W08-S3, although W08-S3 had a 3-bp mutation at the end of its sequence: GTG-CAA (Fig 4).

The amino acid sequence alignment showed that 230 amino acids were encoded by the HJX74 *DTH3*, and only 27 amino acids were encoded by the W08-S3 allele (Fig 5, Table 3). There were few similarities in their sequences, which illustrated a difference in the conformation and structure of DTH3 proteins between these genotypes, likely affecting heading date.

The full length of the *OsDof12* gene was 1323 bp in HJX74 and the secondary SSSLs W05-S2 and W08-S3. There were seven variations in the *OsDof12* sequences between W05-S2 and HJX74, which resulted in three amino acid substitutions (Table 3). The seven nucleotide differences in *OsDof12* between W08-S3 and HJX74 resulted in four amino acid substitutions. An analysis of the physicochemical properties, the hydrophobic and hydrophilic characteristics, and the protein secondary structure revealed that there were no significant differences in the proteins encoded by *OsDof12* among W05-S2, W08-S3, and HJX74, suggesting that changes in this gene were not responsible for the differences in heading date between these lines.

The *Ehd4* gene was 2496 bp long in W05-S2, W08-S3, and HJX74. HJX74 has a 3-bp deletion of nucleotides from 1865 to 1867, whereas W05-S2 and W08-S3 missed three nucleotides at position from 1949 to 1951. In addition, the W05-S2 sequence had 13 other variations compared to HJX74, whereas W08-S3 has 10 (Table 3).

The nucleotide differences between W05-S2, W08-S3, and HJX74 resulted in differences in amino acids of the proteins encoded by *Ehd4*. Each allele contained a total of 831 amino acids; however, HJX74 missed an amino acid, whereas a different amino acid was deleted in W05-S2 and W08-S3. There were two frame shifts between the 621^st^ and 649^th^ amino acid in W08-S3, resulting in major amino acid sequence changes in this region. In addition, there were eight other variations in the *Ehd4* coding sequence in W05-S2, whereas there were additional six variations in the W08-S3 coding sequence.

The analyses of the physicochemical, hydrophobic, and hydrophilic properties, as well as the protein secondary structure, showed that there were no significant differences in the characteristics of *Ehd4* in W05-S2, W08-S3, and HJX74, suggesting that *Ehd4* was not responsible for the changes in heading date in the SSSLs.

### Differentiation of *Hd3a*, *Hd17* and *RFT1* in the SSSLs and the recurrent parent

*Hd3a*, *Hd17* and *RFT1* had been previously mapped to the QTL region flanked by RM587 and RM225. We used secondary SSSL W12-S4 to clone the heading date genes.

**Fig 6:**
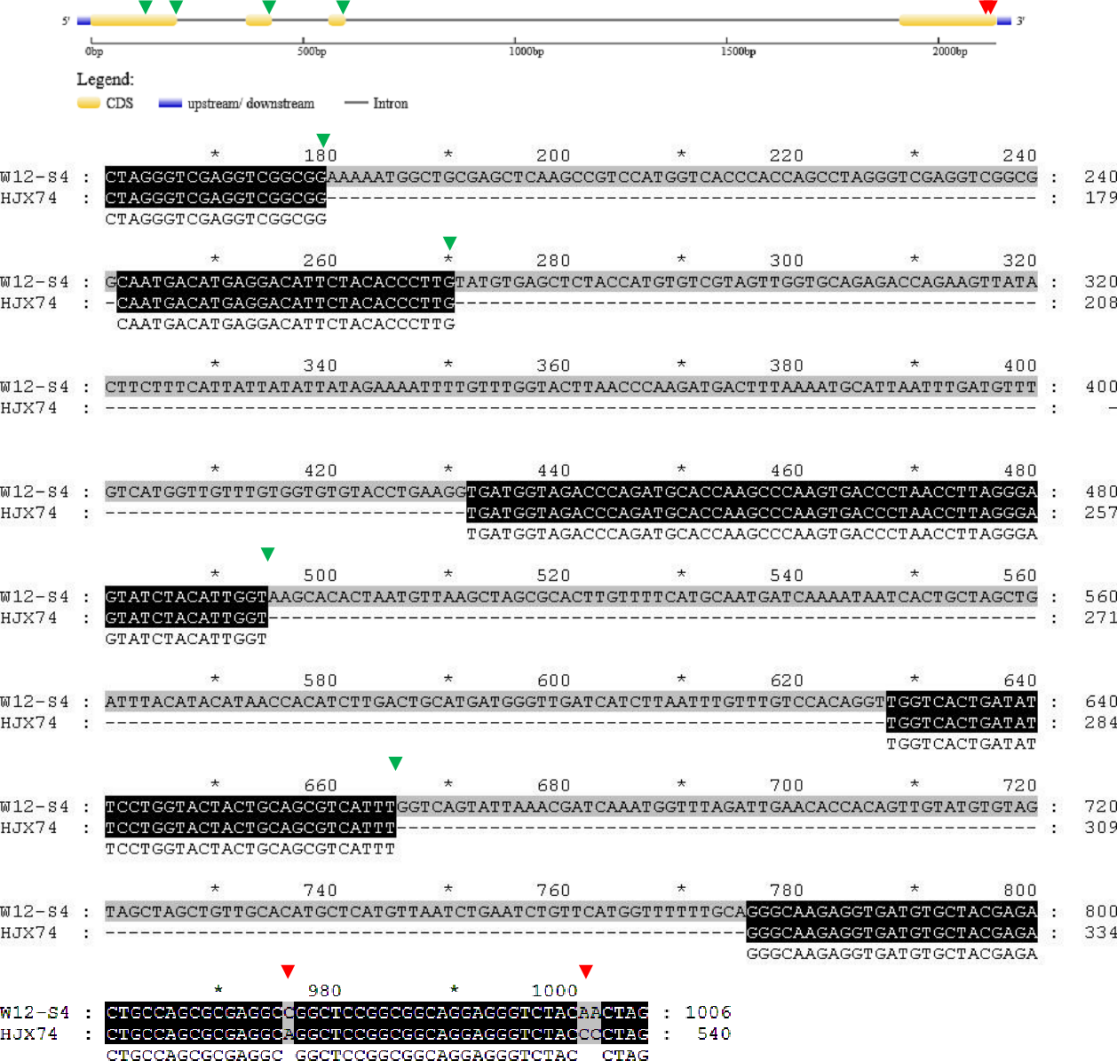
Alignment of *Hd3a* coding sequences from HJX74 and W12-S4. The green triangles indicate the start of deletion/insertion variations between HJX74 and W12-S4, whereas red triangles indicate nucleotide substitutions between HJX74 and W12-S4.

In HJX74, the full CDS length of *Hd3a* was 540 bp; however, in W12-S4, the full CDS length of this gene was 1006 bp. HJX74 missed 466 nucleotides from position 178 to 240, 271 to 431, 495 to 627, and 666 to 775 (Fig 6, Table 3). There were also single nucleotide polymorphisms (SNPs), including an A-C SNP at position 876 and two C-A SNPs at positions 1001 and 1002.

**Fig 7:**
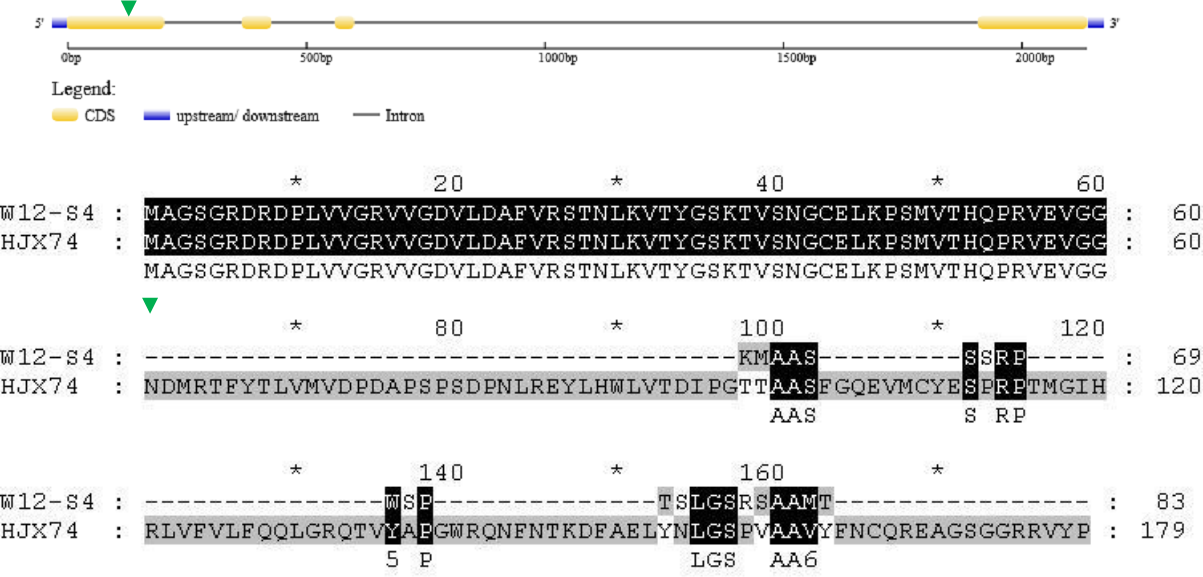
Alignment of Hd3a amino acid sequences from HJX74 and W12-S4. The green triangles indicate amino acid variation between HJX74 and W12-S4.

The nucleotide sequence differences of *Hd3a* between W12-S4 and HJX74 led to changes in the amino acids of protein encoded (Fig 7, Table 3). In HJX74, *Hd3a* encodes 179 amino acids, wheresd in W12-S3, the resulting peptide was just 83 amino acids in length due to the introduction of a premature stop codon into the coding sequence. The first 60 amino acids were identical in W12-S4 and HJX74; however, the major differences in the later amino acid sequence caused a significant difference in the structure and function of the resulting protein. These differences in the Hd3a protein likely resultd in differences in heading dates between these rice lines.

**Fig 8:**
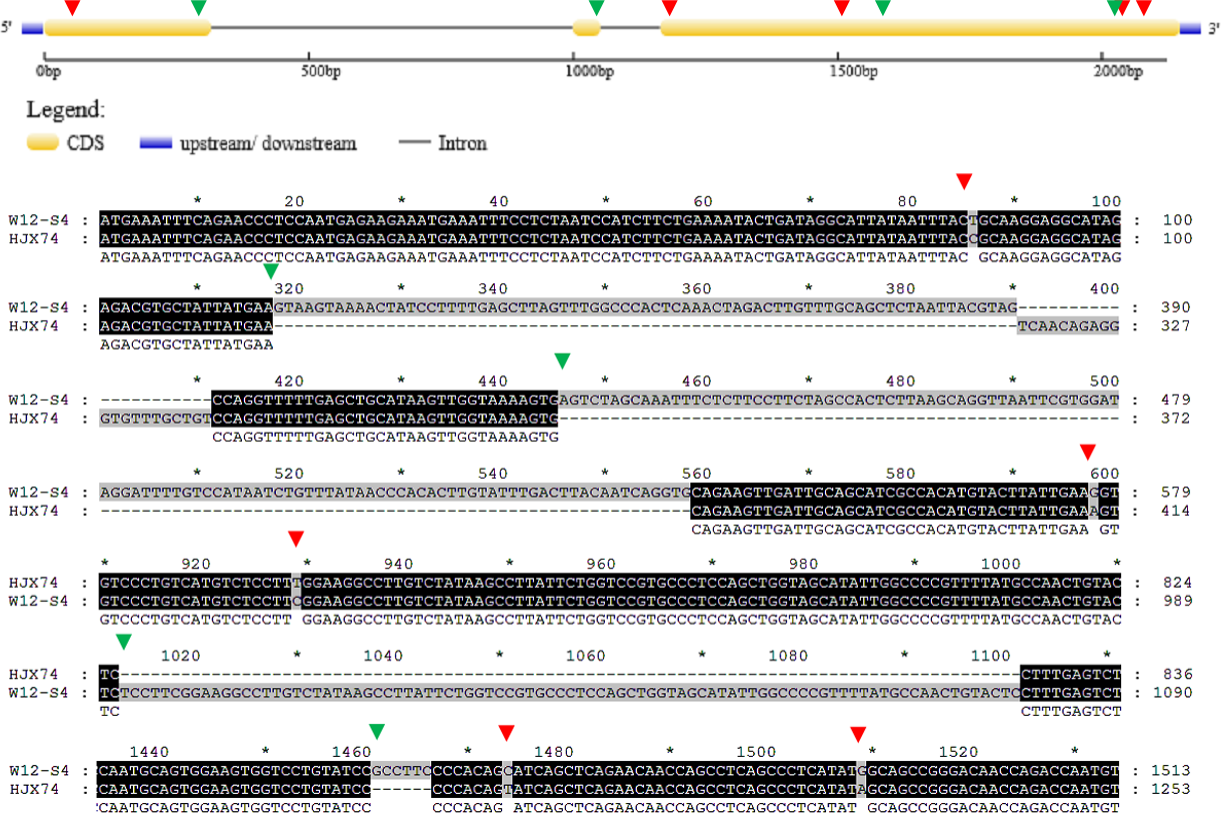
Alignment of *Hd17* coding sequences from HJX74 and W12-S4. The green triangles indicate the start of deletion/insertion variations between HJX74 and W12-S4, whereas red triangles indicate nucleotide substitutions between HJX74 and W12-S4.

**Fig 9:**
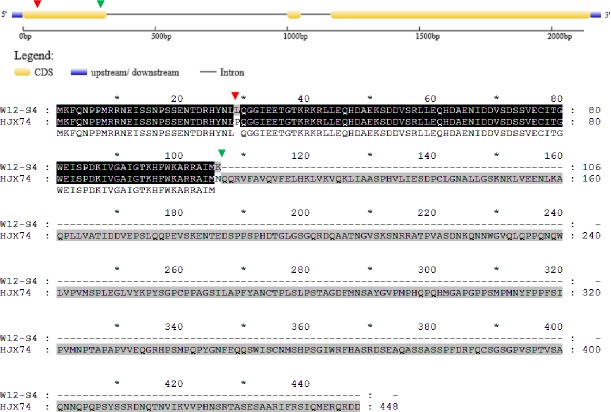
Alignment of Hd17 amino acid sequences from HJX74 and W12-S4. The green triangles indicate the prematurely terminated coding site in W12-S4, whereas red triangles indicate amino acid substitutions between HJX74 and W12-S4.

In HJX74, the full length of *Hd17* was 1358 bp, whereas in W12-S4, it was 1618 bp (Fig 8, Table 3). HJX74 missed a total of 340 base pairs from four positions, which were nucleotides from 340 to 390, 426 to 538, 916 to 1004, and 1440 to 1445, as well as a 21 bp variation at position from 318 to 338. There are a further five SNP variations in the whole *Hd17* coding sequence between HJX74 and W12-S4, including a T-G SNP at position 86, a G-A SNP at position 577, two C-T SNPs at position 908 and 1453, and a C-A SNP at position 1488. Consequently, the amino acid sequences of *Hd17* showed major differences between HJX74 and W12-S4 (Fig 9, Table 3), which likely caused the significant differences in the heading dates of these rice lines.

The full length of *RFT1* in HJX74 and W12-S4 was 537 bp. There were 12 SNP variations in the whole *RFT1* coding sequence between HJX74 and W12-S4 (Table 3), including two T-C SNPs at position 81 and 92, two G-A SNPs at position 313 and 399, three A-G SNPs at position 408, 439 and 478, a G-C SNP at position 411, a C-T SNP at position 420, two G-A SNP at position 431 and 437, and a T-C SNP at position 488. Nucleotide sequence mutation in the *RFT1* resulted in five amino acid mutations (Table 3).

The analyses of the physicochemical, hydrophobic, and hydrophilic properties as well as the protein secondary structure showed that there were no significant differences in the characteristics of *RFT1* in W12-S2 and HJX74, suggesting that *RFT1* was not responsible for the changes in heading date in the SSSLs.

### Differentiation of *Ehd1* and *Ehd2* in the SSSLs and the recurrent parent

*Ehd1* and *Ehd2* had been previously mapped to the QTL region flanked by RM596 and RM269. We used secondary SSSL W22-S2 to clone the heading date gene.

The sequence alignment of *Ehd1* in HJX74 and W22-S2 were completely consistent (Table 3), suggesting that *Ehd1* was not the main reason for the changes in heading date in SSSLs.

**Fig 10:**
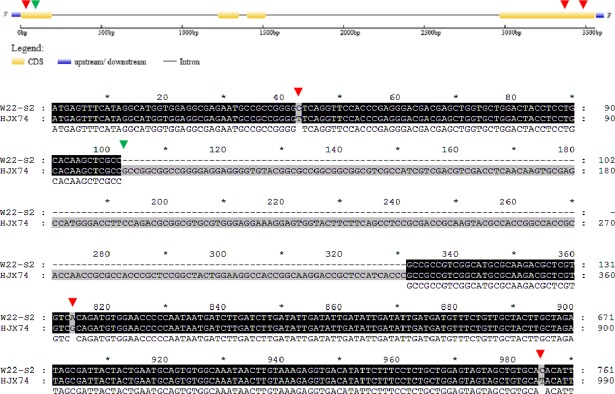
Alignment of *Ehd2* coding sequences from HJX74 and W22-S2. The green triangles indicate the start of deletion/insertion variation between HJX74 and W22-S2, whereas red triangles indicate nucleotide substitutions between HJX74 and W22-S2.

In HJX74, the full length of *Ehd2* was 1045 bp, whereas in the SSSL W22-S2, the full length of *Ehd2* was 816 bp. As show in Fig 10, W22-S2 had a deletion of 229 nucleotides between 103 and 331 positions (Table 3). Moreover, when compared HJX74 with W22-S2, there were two C-T SNPs at the 43^rd^ and 985^th^ nucleotide, and an A-G SNP at position 814.

**Fig 11:**
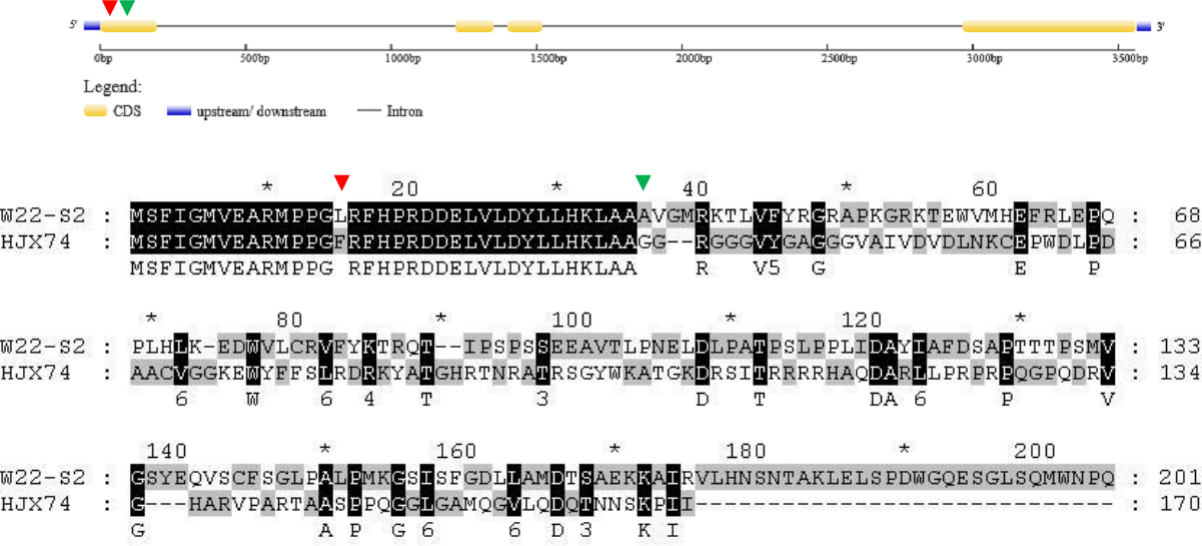
Alignment of Ehd2 amino acid sequences from HJX74 and W22-S2. The green triangles indicate the start site of frameshift in W12-S4, whereas red triangles indicate amino acid substitutions between HJX74 and W12-S4.

The proteins encoded by the *Ehd2* genes in W22-S2 and HJX74 had major differences in their amino acid sequences (Fig 11, Table 3). *EHD2* had 170 amino acids in HJX74, but 201 in W22-S2; the longer polypeptide chain was caused by a delayed stop codon which was resulted by frameshift. The different amino acid sequences of *EHD2* between HJX74 and W22-S2 might cause the significant changes in the heading date in SSSLs.

## Discussion

Genome sequencing projects have been completed for several plant species and many more projects for sequencing new genomes are underway. To exploit the potential of this resource, researchers have shifted attention to a variety of functional genomic tools that can help decipher the functions of thousands of newly identified genes [34]. The definition of gene functions requires the phenotypic characterization of genetic variation, including naturally occurring variation among accessions; however, the multigenic nature of this variation has limited its application [35]. With the development of the secondary mapping populations, exploitation of the naturally occurring variation will be feasible and become more systematic and efficient.

The mapping populations comprised of SSSLs could enable the exploitation of naturally occurring variation. Eshed and Zamir [30] developed a population consisting of 50 introgression lines, each containing a single homozygous chromosome segment from the wild tomato (*Solanum pennellii*) in the genetic background of a tomato cultivar (*S*. *lycopersicum*). Recently, a library of 1,123 SSSLs has been constructed in rice, using the cultivar HJX74 as the recipient (background) parent and 24 other varieties as donors of substituted chromosome segments, which had an average of 19.3 cM in size [20]. With a high level of uniformity in the genetic background, the SSSLs have been used to detect QTLs by direct comparison with the recipient parent [11, 21, 22]. In the present study, we investigated four SSSLs with different heading dates to dissect the QTLs involved in its regulation. A QTL could contain a single gene of interest or a cluster of genes involved in the same process. Therefore, using secondary SSSLs derived from the four primary SSSLs, we narrowed down the size of the heading date QTLs on chromosomes 3, 6, and 10. In addition, we were able to dissect a heading date QTL, detected originally on a 10.4-cM substituted segment on chromosome 6 in SSSL W12, into two tightly linked QTLs, *qHD-6-1* and *qHD-6-2*, for a total of four heading date QTLs identified in this study. These results demonstrated that genetic dissection using SSSLs was a powerful tool for detecting and investigating QTLs for complex traits.

Many genes controlling Mendelian traits have been identified in the past 20 years; however, relatively few genes underlying genetically complex traits have been reported. Genes that contribute to complex traits or QTLs pose special challenges that make their discovery more difficult [36]. Many important traits in rice, including heading date, plant height, panicle size, number of grains per spike, and the number of tillers are controlled by quantitative trait loci (QTLs) and show continuous phenotypic variation in progenies. Construction and use of a suitable genetic population has obvious advantages for fine mapping of QTLs and map-based cloning of the target genes.

For the map-based cloning of a QTL, the first step is to identify the chromosome region on which it is located [37]. In the present study, four QTLs for heading date in rice were mapped to small intervals without the costly and time-consuming production of a large experimental population. This demonstrated that the development of SSSLs with small substituted segments made mapping QTLs for complex traits more systematicly and efficiently, which enhanced our ability to separate clusters of genes into a single Mendelian factor and simplified the discovery of genes underlying genetically complex traits.

Heading date is an important agronomic trait in rice; it also determines the geographical and seasonal adaptability of this crop. For the four identified QTLs, we analyzed the alleles of eight known heading date genes, which were located within the regions on chromosome 3 (*DTH3*, *OsDof12*, and *Ehd4*), chromosome 6 (*Hd3a*, *RFT1* and *Hd17*), and chromosome 10 (*Ehd1* and *Ehd2*), and showed differention between HJX74 and SSSL. We speculated that the genetic differentiation of *DTH3*, *Hd17* and/or *Hd3a*, and *Ehd2* would play important roles in heading date variation of W05-S2, W08-S3, W12-S4 and W22-S2 respectively, even though the involvement of other genes might not be ruled out.

*DTH3*, an important early flowering gene in long day and short day conditions, can significantly up-regulate the expression of the early flowering gene *Ehd1*. *Ehd1* can further promote the expression of *RFT1* gene. Thus *DTH3* is an important catalytic factor of rice flowering [38]. Under the conditions of long day, overexpression of *OsDof12* leads to the early heading, but it has no effect on the heading date under the short-day conditions. *OsDof12* promotes the heading date of rice by increasing the expression of *Hd3a* and *OsMADS14* under the long-day conditions [39].

In the present study, the heading dates of HJX74, W05-S2, and W08-S3 were 105±0.59, 124.45±0.89, and 94.23±0.78, respectively. Relative to HJX74 W05-S2 showed late heading, whereas W08-S3 showed early heading.

We cloned three different alleles *DTH3*, *OsDof12*, and *Ehd4*, using secondary substitution lines W05-S2 and W08-S3. Both the coding sequences of *OsDof12* and *Ehd4* and the amino acid sequences they encoded showed little difference among W05-S2, W08-S3, and HJX74, we speculated that these genes might not be responsible for the phenotypic changes of heading date.

On the other hand, the coding sequences *DTH3* and the amino acid sequences in encodes are significantly different between W05-S2, W08-S3, and HJX74; we speculate that the late heading of W05-S2 and the early heading of W08-S3 are due to the genetic differentiation of the *DTH3* allele even if other genes do not rule out the possibility of functioning.

Natural variation in *Hd17* may change the transcription level of a flowering repressor *Ghd7*, suggesting that *Hd17* is involved in the photoperiodic flowering pathway [3]. *Hd3a* is the main promoter of floral transition and flowering at about 60 DAS under SD conditions in wild-type plants [23]. *RFT1* is the closest homologue of *Hd3a*, and is thought to encode a mobile flowering signal and promotes floral transition under short-day (SD) conditions [40].

Substitution line W12-S4 showed late heading compared to HJX74, and 3 heading date genes *Hd17*, *Hd3a*, and *RFT1*, have been delimited in the substituted interval. The coding sequences of *RFT1* showed high similarity between HJX74 and W12-S4, whereas the coding sequences of *Hd17* and *Hd3a* genes and the protein secondary structure of these two proteins were very different between HJX74 and W12-S4. Therefore, we speculated that probably one or two of the *Hd17* and *Hd3a* resulted in the late heading.

Expression analysis of the genes related to rice flowering in the *ehd2* mutant and the wild type has revealed genetic interactions between *Hd1* and *Ehd2*, which demonstrate that *Ehd2* promotes floral transition mainly by up-regulating *Ehd1* and genes downstream of *Ehd1*, such as *Hd3a* and *RFT1* [41].

In the present study, W22-S2 showed late heading compared to HJX74, and heading date genes *Ehd1* and *Ehd2* have been cloned in this substituted interval. There was no difference between HJX74 and W22-S2 in the CDS of *Ehd1*, while *Ehd2*, both in nucleotide and amino acid sequences showed significantly difference. So we speculated that the difference of *Ehd2* between HJX74 and W22-S2 might be the cause of the late heading.

The recurrent parent HJX74 and four donor rice accessions of SSSLs are stored in the China National Crop Gene Bank (CNCGB) in the Institute of Crop Sciences, Chinese Academy of Agricultural Sciences (CAAS) (S2 Table). The genome sequences in the present study were compared with those stored in the databases of 3000 resequenced rice lines on SNPSEEK [42], and the results were shown in S3 Table. The comparison showed that there are differences in genome sequences, and we believed that these differences were the individual differences among the samples. Although we have done our best, there was no guarantee that the sequences were always consistent with those of the literature. Nonetheless, our sequencing reports were the true results of the sample sequences.

With the completion of the sequences of rice chromosomes, a detail comparison of the chromosomal location of QTLs in this work with those cloned or mapped genes (QTLs) can be performed by BLASTN analysis against the sequence of the whole rice genome. On the other hand, we have developed a large secondary segregating population using target substitution lines to locate the heading genes in this substitution lines and to prove which genes play a key role in the substitution lines.

The mRNA transcripts of genes are not always intact. Additionally, the reverse transcriptase used in cDNA synthesis often shows low fidelity. To evaluate the soundness of the results (Figs 4–11 and Table 3), we sequenced the genomic DNAs of the candidate genes and compared them with the cDNAs of the candidate genes (S1–S8 Figs), which further confirmed the accuracy of the results in this article. In addition, the genomic DNA or the cDNA of each candidate gene were sequenced six times, and sequence consistency was guaranteed.

Taken together, this study illustrated that genetic variations of the heading date genes identified in these QTLs would serve as useful resources for the further study on gene function and evolution, as well as for the selection of valuable genes in rice breeding. We have also shown that secondary SSSLs were powerful tools for the genetic dissection and allelic polymorphisms in genes.

## Supporting information

S1 Table Primer sequences used in this study. (DOC)

S2 Table Information for the recurrent parent and four donor rice accessions of SSSL. (DOCX)

S3 Table Sequence discrepancy between this study and SNPSEEK. (DOCX)

S1 Fig Comparative analysis of genomic DNA and cDNA of *DTH3* gene. (DOC)

S2 Fig Comparative analysis of genomic DNA and cDNA of *EHD1* gene. (DOC)

S3 Fig Comparative analysis of genomic DNA and cDNA of *EHD2* gene. (DOC)

S4 Fig Comparative analysis of genomic DNA and cDNA of *EHD4* gene. (DOC)

S5 Fig Comparative analysis of genomic DNA and cDNA of *Hd3a* gene. (DOC)

S6 Fig Comparative analysis of genomic DNA and cDNA of *Hd17* gene. (DOC)

S7 Fig Comparative analysis of genomic DNA and cDNA of *OsDof12* gene. (DOC)

S8 Fig Comparative analysis of genomic DNA and cDNA of *RFT1* gene. (DOC)

## References

[1] Fitzgerald MA, McCouch SR, Hall RD. Not just a grain of rice: The quest for quality. Trends plant sci. 2009; 14(3): 133–139. doi: 10.1016/j.tplants.2008.12.004 19230745 10.1016/j.tplants.2008.12.004

[2] Gao H, Jin MN, Zheng XM, Chen J, Yuan DY, Xin YY, et al *Days to heading 7*, a major quantitative locus determining photoperiod sensitivity and regional adaptation in rice. Proc Natl Acad Sci USA. 2014; 111(46): 16337–16342. doi: 10.1073/pnas.1418204111 25378698 10.1073/pnas.1418204111 PMC4246261

[3] Matsubara K, Ogiso-Tanaka E, Hori K, Ebana K, Ando T, Yano M. Natural variation in *Hd17*, a homolog of *Arabidopsis ELF3* that is involved in rice photoperiodic flowering. Plant Cell Physiol. 2012; 53(4), 709–716. doi: 10.1093/pcp/pcs028 22399582 10.1093/pcp/pcs028

[4] Gao H, Zheng XM, Fei GL, Chen J, Jin MN, Ren YL, et al *Ehd4* encodes a novel and Oryza-genus-specific regulator of photoperiodic flowering in rice. PLoS Genet. 2013; 9(2):e1003281 doi: 10.1371/journal.pgen.1003281 23437005 10.1371/journal.pgen.1003281 PMC3578780

[5] Hori K, Ogiso-Tanaka E, Matsubara K, Yamanouchi U, Ebana K, Yano M. *Hd16*, a gene for casein kinase I, is involved in the control of rice flowering time by modulating the day-length response. Plant J. 2013; 76(1), 36–46. doi: 10.1111/tpj.12268 23789941 10.1111/tpj.12268 PMC4223384

[6] Wu WX, Zheng XM, Lu GW, Zhong ZZ, Gao H, Chen LP, et al Association of functional nucleotide polymorphisms at DTH2 with the northward expansion of rice cultivation in Asia. Proc Natl Acad Sci USA. 2013; 110(8): 2775–2780. doi: 10.1073/pnas.1213962110 23388640 10.1073/pnas.1213962110 PMC3581972

[7] Doi K, Izawa T, Fuse T, Yamanouchi U, Kubo T, Shimatani Z, et al *Ehd1*, a B-type response regulator in rice, confers short-day promotion of flowering and controls FT-like gene expression independently of *Hd1*. Genes Dev. 2004; 18:926–936. doi: 10.1101/gad.1189604 15078816 10.1101/gad.1189604 PMC395851

[8] Kojima S, Takahashi Y, Kobayashi Y, Monna L, Sasaki T, Arak T, et al *Hd3a*, a rice ortholog of the *Arabidopsis* FT gene, promotes transition to flowering downstream of *Hd1* under short-day conditions. Plant Cell Physiol. 2002; 43(10):1096–1105. 12407188 10.1093/pcp/pcf156

[9] Yano M, Sasaki T. Genetic and molecular dissection of quantitative traits in rice. Plant Mol Biol. 1997; 35: 145–153. 9291968

[10] Yamamoto T, Lin HX, Sasaki T, Yano M. Identification of heading date quantitative trait locus *Hd6*, and characterization of its epistatic interaction with *Hd2* in rice using advanced backcross progeny. Genetics. 2000; 154: 885–891. 10655238 10.1093/genetics/154.2.885 PMC1460948

[11] Eshed Y, Zamir D. An introgression line population of *Lycopersicon pennellii* in the cultivated tomato enables the identification and fine mapping of yield-associated QTL. Genetics. 1995; 141: 1147–1162. 8582620 10.1093/genetics/141.3.1147 PMC1206837

[12] Howell PM, Marshall DF, Lydiate DJ. Towards developing inter-varietal substitution lines in *Brassica napus* using marker-assisted selection. Genome. 1996; 39: 348–358 18469898 10.1139/g96-045

[13] Koumproglou R, Wilkes TM, Townson P, Wang XY, Beynon J, Pooni HS, et al STAIRS: a new genetic resource for functional genomic studies of *Arabidopsis*. Plant J. 2002; 31: 355–364. 12164814 10.1046/j.1365-313x.2002.01353.x

[14] Sobrizal K, Ikeda K, Sanchez PL, Doi K, Angeles ER, Khush GS, et al Development of *Oryza glumaepatula* introgression lines in rice, *O*. *sativa* L. Rice Genet Newslett. 1999; 16:107–108.

[15] Kubo T, Aida Y, Nakamura K, Tsunematsu H, Doi K, Yoshimura A. Reciprocal chromosomal segment substitution series derived from *Japonic* and *Indica* cross of rice (*Oryza sativa* L.). Breeding Sci. 2002; 52: 319–325.

[16] Yano M. Genetic and molecular dissection of naturally occurring variation. Curr opin plant biol. 2001; 4(2): 130–135. 11228435 10.1016/s1369-5266(00)00148-5

[17] He FH, Xi ZY, Zeng RZ, Talukdar A, Zhang GQ. Mapping of heading date QTLs in rice (*Oryza sativa* L.) using single segment substitution lines. Acta Agric Sin. (2005); 38: 1505–1513. 16231737

[18] Zhu HT, Liu ZQ, Fu XL, Dai ZJ, Wang SK, Zhang GQ, et al Detection and characterization of epistasis between QTLs on plant height in rice using single segment substitution lines. Breeding Sci. 2015; 65(3): 192–200. 10.1270/jsbbs.65.192 PMC4482168 26175615

[19] Ebitani T, Takeuchi Y, Nonoue Y, Yamamoto T, Takeuchi K, Yano M. Construction and evaluation of chromosome segment substitution lines carrying overlapping chromosome segments of *indica* rice cultivar ‘Kasalath’ in a genetic background of *japonica* elite cultivar ‘Koshihikari’. Breeding Sci. 2005; 55:65–73.

[20] Zhang GQ, Zeng RZ, Zhang ZM, Ding XH, Li WT, Liu GM, et al The construction of a library of single segment substitution lines in rice (*Oryza sativa* L.). Rice Genet Newslett. 2004; 21:85–87.

[21] Zhao FM, Zhu HT, Ding XH, Zeng RZ, Zhang ZM, Li WT, et al Detection of QTLs for important agronomic traits and analysis of their stabilities using SSSLs in rice. Agr Sci China. 2007; 6(7):769–778.

[22] Xi ZY, He FH, Zeng RZ, Zhang ZM, Ding XH, Li WT, et al Development of a wide population of chromosome single segment substitution lines (SSSLs) in the genetic background of an elite cultivar in rice (*Oryza sativa* L.). Genome. 2006; 49(5): 476–484. doi: 10.1139/g06-005 16767172 10.1139/g06-005

[23] Monna L, Lin HX, Kojima S, Sasaki T, Yano M. Genetic dissection of a genomic region for a quantitative trait locus, *Hd3*, into two loci, *Hd3a* and *Hd3b*, controlling heading date in rice. Theor Appl Genet. 2002; 104: 772–778. doi: 10.1007/s00122-001-0813-0 12582636 10.1007/s00122-001-0813-0

[24] Zheng KL, Huang N, Bennett J, Khush GS. PCR-based marker-assisted selection in rice breeding IRRI discussion paper series. NO.12. IRRI, Manila, Philippines 1995.

[25] Panaud O, Chen X, McCouch SR. Development of microsatellite markers and characterization of simple sequence length polymorphism (SSLP) in rice (*Oryza sativa* L). Mol Gen Genet. 1996; 252:597–607. 8914521 10.1007/BF02172406

[26] Li WT, Zeng RZ, Zhang ZM, Zhang GQ. Mapping of *S-b* locus for F_1_ pollen sterility in cultivated rice using PCR based markers. Acta Bot Sin. 2002; 44 (4):463–467.

[27] McCouch SR, Teytelman L, Xu Y, Lobos KB, Clar K, Walton M, et al Development and Mapping of 2240 New SSR Markers for Rice (*Oryza sativa* L.). DNA Res. 2002; 9:199–207. 12597276 10.1093/dnares/9.6.199

[28] Huang CF. Development of position-specific microsatellite marker and mapping of insect resistant gene in rice. M Sc. thesis submitted to the South China Agricultural University, Guangzhou, China. 2003.

[29] Wissuwa M, Wegner J, Ae N, Yano M. Substitution mapping of *Pup1*: a major QTL increasing phosphorus uptake of rice from a phosphorus-deficient soil. Theor Appl Genet. 2002; 105: 890–897. doi: 10.1007/s00122-002-1051-9 12582914 10.1007/s00122-002-1051-9

[30] Eshed Y, Zamir D. A genomic library of *Lycopersicon pennellii* in *L*.*esculentum*: A tool for fine mapping of genes. Euphytica. 1994; 79:175–179.

[31] Larkin MA, Blackshields G, Brown NP, Chenna R, McGettigan PA, McWilliam H, et al Clustal W and Clustal X version 2.0. Bioinformatics. 2007; 23(21), 2947–2948. doi: 10.1093/bioinformatics/btm404 17846036 10.1093/bioinformatics/btm404

[32] Nicholas KB: GeneDoc: Analysis and Visualization of Genetic Variation. EMBNET NEWS. 1997, 4: 14.

[33] Hu B, Jin JP, Guo AY, Zhang H, Luo JC, Gao G. GSDS 2.0: an upgraded gene feature visualization server. Bioinformatics, 2015; 31(8):1296–1297. doi: 10.1093/bioinformatics/btu817 25504850 10.1093/bioinformatics/btu817 PMC4393523

[34] Gilchrist EJ, Haughn GW. TILLING without a plough: a new method with applications for reverse genetics. Curr Opin Plant Biol. 2005; 8:211–215. doi: 10.1016/j.pbi.2005.01.004 15753003 10.1016/j.pbi.2005.01.004

[35] Alonso-Blanco C, Koornneef M. Naturally occurring variation in *Arabidopsis*: an underexploited resource for plant genetics. Trends Plant Sci. 2000; 5:22–29. 10637658 10.1016/s1360-1385(99)01510-1

[36] Glazier AM, Nadeau JH, Aitman TJ. Finding genes that underlie complex traits. Science. 2002; 298:2345–2349. doi: 10.1126/science.1076641 12493905 10.1126/science.1076641

[37] Yano M, Katayose Y, Ashikari M, Yamanouchi U, Monna L, Fuse T, et al *Hd1*, a major photoperiod sensitivity quantitative trait locus in rice, is closely related to the *Arabidopsis* flowering time gene *CONSTANS*. Plant Cell. 2000; 12(12): 2473–2483. 11148291 10.1105/tpc.12.12.2473 PMC102231

[38] Bian X F, Liu X, Zhao Z G, Jiang L, Gao H, Zhang YH, et al Heading date gene, *dth3* controlled late flowering in *O*. *Glaberrima Steud*. by down-regulating *Ehd1*. Plant Cell Rep. 2011; 30: 2243–2254. doi: 10.1007/s00299-011-1129-4 21830130 10.1007/s00299-011-1129-4

[39] Li DJ, Yang CH, Li XB, Ji GB, Zhu LH. Sense and antisense *OsDof12* transcripts in rice. BMC mol biol. 2008; 9(1): 80. 18796165 10.1186/1471-2199-9-80 PMC2576344

[40] Komiya R, Yokoi S, Shimamoto K. A gene network for long-day flowering activates *RFT1* encoding a mobile flowering signal in rice. Development. 2009; 136(20): 3443–3450. doi: 10.1242/dev.040170 19762423 10.1242/dev.040170

[41] Matsubara K, Yamanouchi U, Wang ZX, Minobe Y, Izawa T, Yano M. *Ehd2*, a rice ortholog of the maize *INDETERMINATE1* gene, promotes flowering by up-regulating *Ehd1*. Plant Physiol. 2008; 148(3): 1425–1435. doi: 10.1104/pp.108.125542 18790997 10.1104/pp.108.125542 PMC2577255

[42] Li ZK, Fu BY, Gao YM, Wang WS, Xu JL, Zhang F, et al The 3,000 rice genomes project. GigaScience. 2014 3:7 doi: 10.1186/2047-217X-3-7 24872877 10.1186/2047-217X-3-7 PMC4035669

