# Supplementary material for: Genetic dissection of QTLs and differentiation analysis of alleles for heading date genes in rice
Source: PLoS One. 2018 Jan 3;13(1):e0190491. doi: 10.1371/journal.pone.0190491 (PMC5752018; doi:10.1371/journal.pone.0190491)
Supplement: S1 Table — (DOC) [file pone.0190491.s001.doc]

**S1 Table** Primer sequences used in this study

| **Primer** | **Sequence** (5'---3') |
| --- | --- |
| *DTH3*-F | ATCCCCTTGCGTTTTACCACTTTTG |
| *DTH3*-R | AGACCCCCGTTCCCCACCAC |
| *Ehd2*-F | GAGATGAGTTTCATAGGCATGGTG |
| *Ehd2*-R | CAAGCCATTTTCACTACTCTACTGTG |
| *Ehd4*-F | GGACGATGATCCAATCGGTAA |
| *Ehd4*-R | CAGTGCCAAATCCATCAGACC |
| *Hd3a*-F1 | TAGCTGCCTCTATCACAGTATATTTGC |
| *Hd3a*-R1 | TCATGTCATTGCCGCCGAC |
| *Hd17*-F1 | CACACAAAAGCCCTTCAACAG |
| *Hd17*-R1 | TTTATTCATCAAAGCTACCTATACGT |
| *OsDof12*-F | GCTTGTGCGAATGAAAAGATT |
| *OsDof12*-R | GGACTGGTTTAATACACTGGAATC |
