# Supplementary material for: Genetic dissection of QTLs and differentiation analysis of alleles for heading date genes in rice
Source: PLoS One. 2018 Jan 3;13(1):e0190491. doi: 10.1371/journal.pone.0190491 (PMC5752018; doi:10.1371/journal.pone.0190491)
Supplement: S2 Table — (DOCX) [file pone.0190491.s002.docx]

**S2 Table** Information for the recurrent parent and four donor rice accessions of SSSL.

| Variety name | Origin country | Variety group | Seed source |
| --- | --- | --- | --- |
| Huagengxian 74 | China | *Indica* | CNCGB**^1^** |
| Zihui 100 | China | *Indica* | CNCGB |
| Khazar | Iran | *Indica* | CNCGB |
| IR64a | Phillipines | *Indica* | CNCGB |
| IR58025B | Phillipines | *Indica* | CNCGB |

^1^ CNCGB: the China National Crop Gene Bank (CNCGB) in the Institute of Crop Sciences, Chinese Academy of Agricultural Sciences (CAAS).
