# Supplementary material for: Genetic dissection of QTLs and differentiation analysis of alleles for heading date genes in rice
Source: PLoS One. 2018 Jan 3;13(1):e0190491. doi: 10.1371/journal.pone.0190491 (PMC5752018; doi:10.1371/journal.pone.0190491)
Supplement: S3 Table — (DOCX) [file pone.0190491.s003.docx]

**S3 Table** Sequence discrepancy between this study and SNPSEEK

| SSSL | Donor | Causal gene | Location | This study | SNPSEEK |
| --- | --- | --- | --- | --- | --- |
| W05-S2 | Zihui 100 | *DTH3* | 59-end | Deletion | - |
|  |  | *OsDof12* | 71 | C | T |
|  |  |  | 769 | G | A |
|  |  | *Ehd4* | 717 | T | C |
|  |  |  | 1169 | G | A |
|  |  |  | 1865-1868 | Deletion | - |
|  |  |  | 1912 | G | A |
|  |  |  | 2000 | G | T |
|  |  |  | 2147 | T | C |
| W08-S3 | IR64a | *DTH3* | 78-79 | AG | GT |
|  |  |  | 82-end | Deletion | - |
|  |  | *OsDof12* | 219 | T | C |
|  |  |  | 850 | G | A |
|  |  |  | 882 | T | A |
|  |  | *Ehd4* | 652 | G | C |
|  |  |  | 1150 | T | A |
|  |  |  | 1550 | T | C |
| W12-S4 | IR58025B | *Hd3a* | 761 | T | C |
|  |  | *Hd17* | 86 | T insert | - |
|  |  |  | 389 | G | T |
|  |  | *RFT1* | 399 | A | G |
|  |  |  | 408 | G | A |
|  |  |  | 411 | C | G |
|  |  |  | 420 | T | C |
|  |  |  | 429 | A | G |
|  |  |  | 438 | A | G |
|  |  |  | 444 | A | G |
|  |  |  | 446 | G | A |
|  |  |  | 485 | G | A |
|  |  |  | 495 | C | T |
| W22-S2 | Khazar | *Ehd1* | 134 | G | A |
|  |  |  | 187 | C | T |
|  |  |  | 204 | T | A |
|  |  |  | 248-268 | - | Deletion |
|  |  |  | 455 |  | A |
|  |  |  | 594 | T | C |
|  |  |  | 674 | G | A |
|  |  | *Ehd2* | - | - | - |
