# Supplementary figures and images for: Genetic dissection of QTLs and differentiation analysis of alleles for heading date genes in rice
Source: PLoS One. 2018 Jan 3;13(1):e0190491. doi: 10.1371/journal.pone.0190491 (PMC5752018; doi:10.1371/journal.pone.0190491)

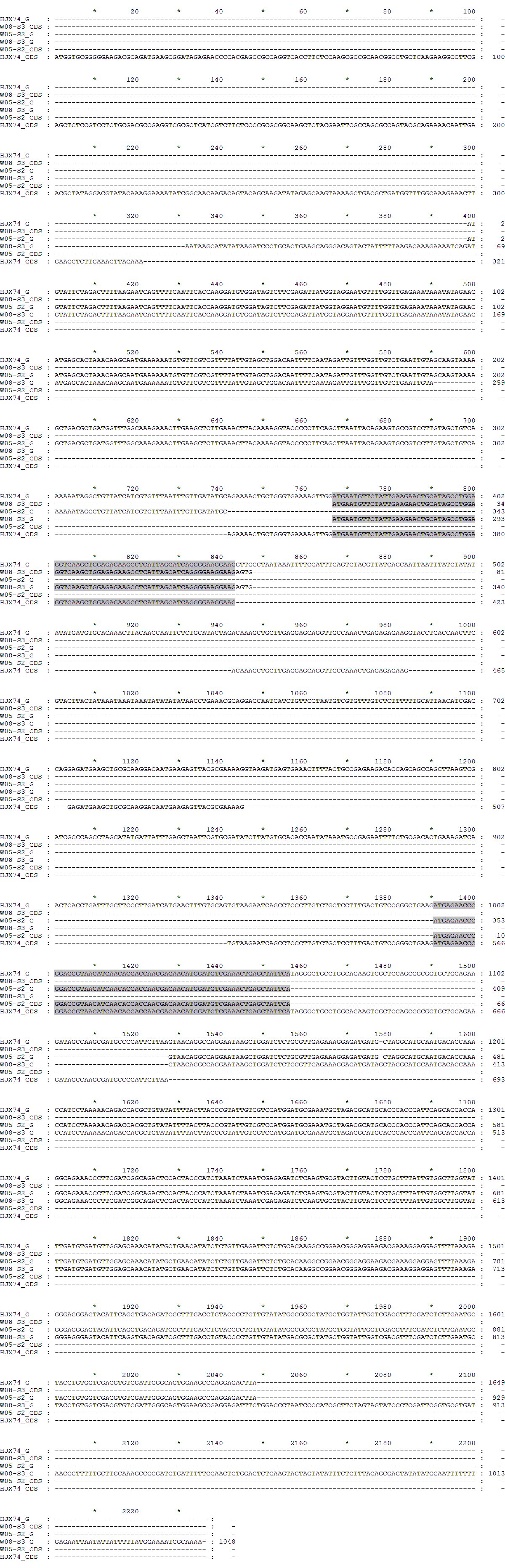
S1 Fig. Comparative analysis of genomic DNA and cDNA of *DTH3* gene.

Supplement: S1 Fig — (DOC) [file pone.0190491.s004.doc]

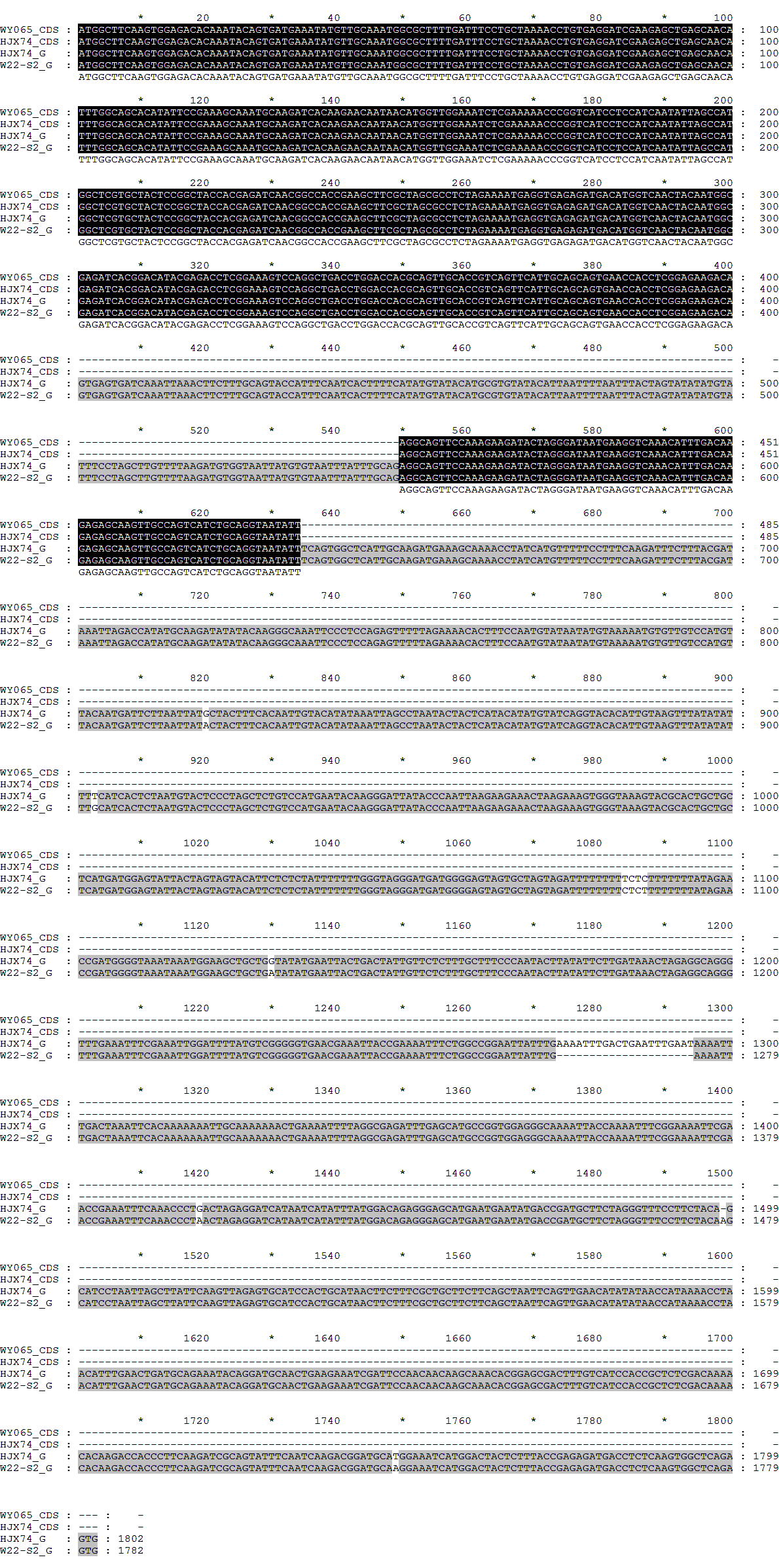


S2 Fig. Comparative analysis of genomic DNA and cDNA of *EHD1* gene.

Supplement: S2 Fig — (DOC) [file pone.0190491.s005.doc]

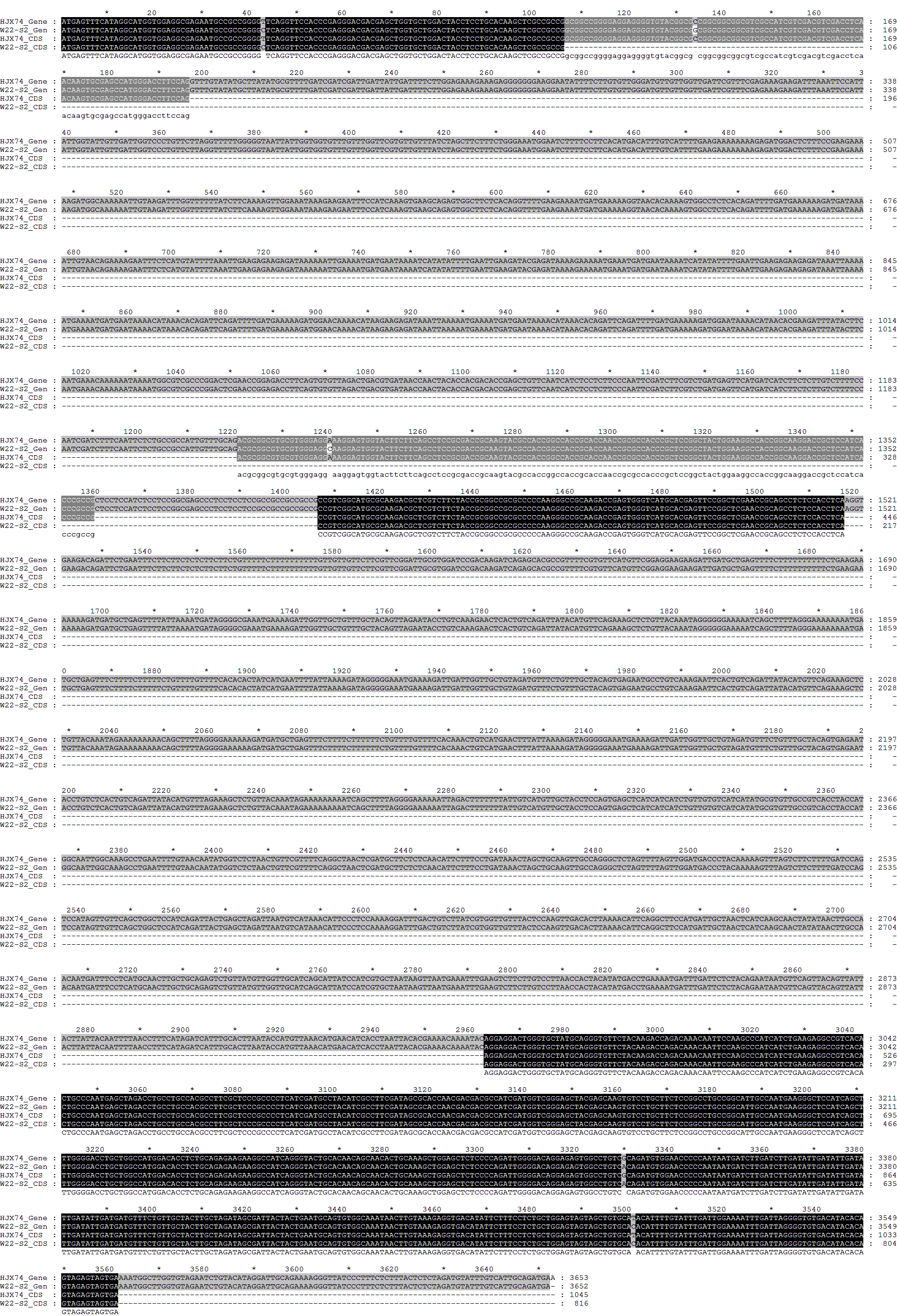


S3 Fig. Comparative analysis of genomic DNA and cDNA of *EDH2* gene.

Supplement: S3 Fig — (DOC) [file pone.0190491.s006.doc]

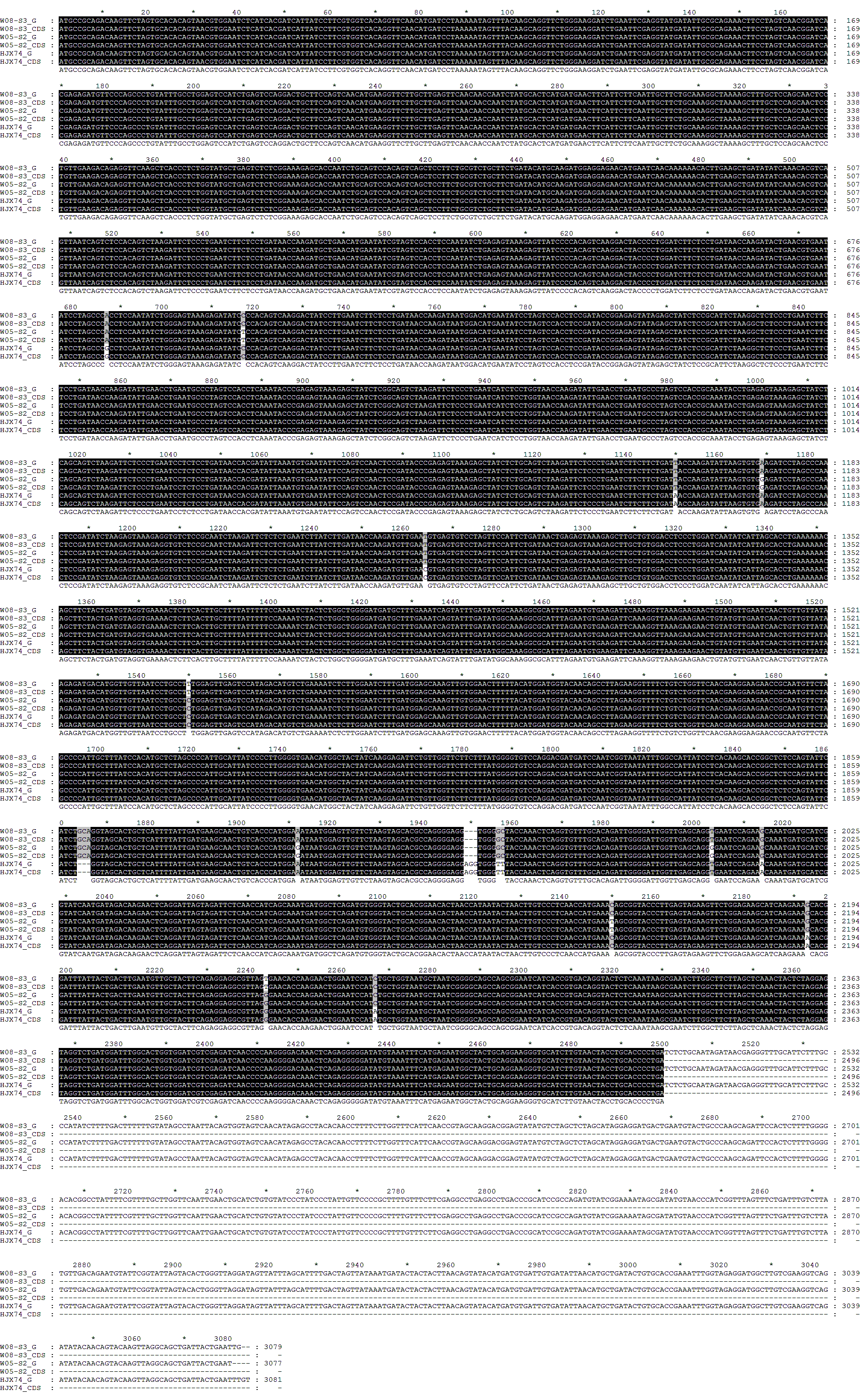
S4 Fig. Comparative analysis of genomic DNA and cDNA of *EHD4* gene.

Supplement: S4 Fig — (DOC) [file pone.0190491.s007.doc]

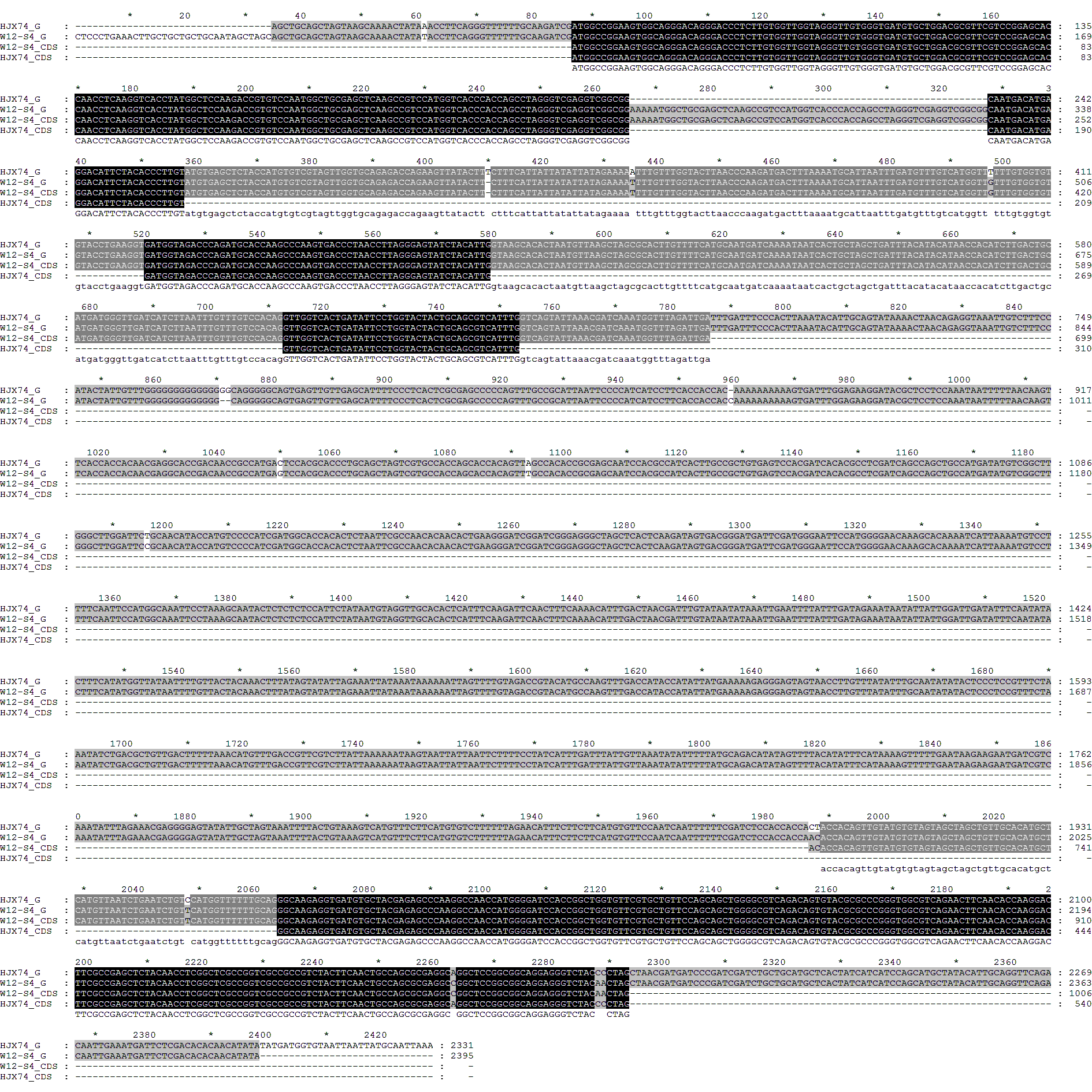


S5 Fig. Comparative analysis of genomic DNA and cDNA of *Hd3a* gene.

Supplement: S5 Fig — (DOC) [file pone.0190491.s008.doc]

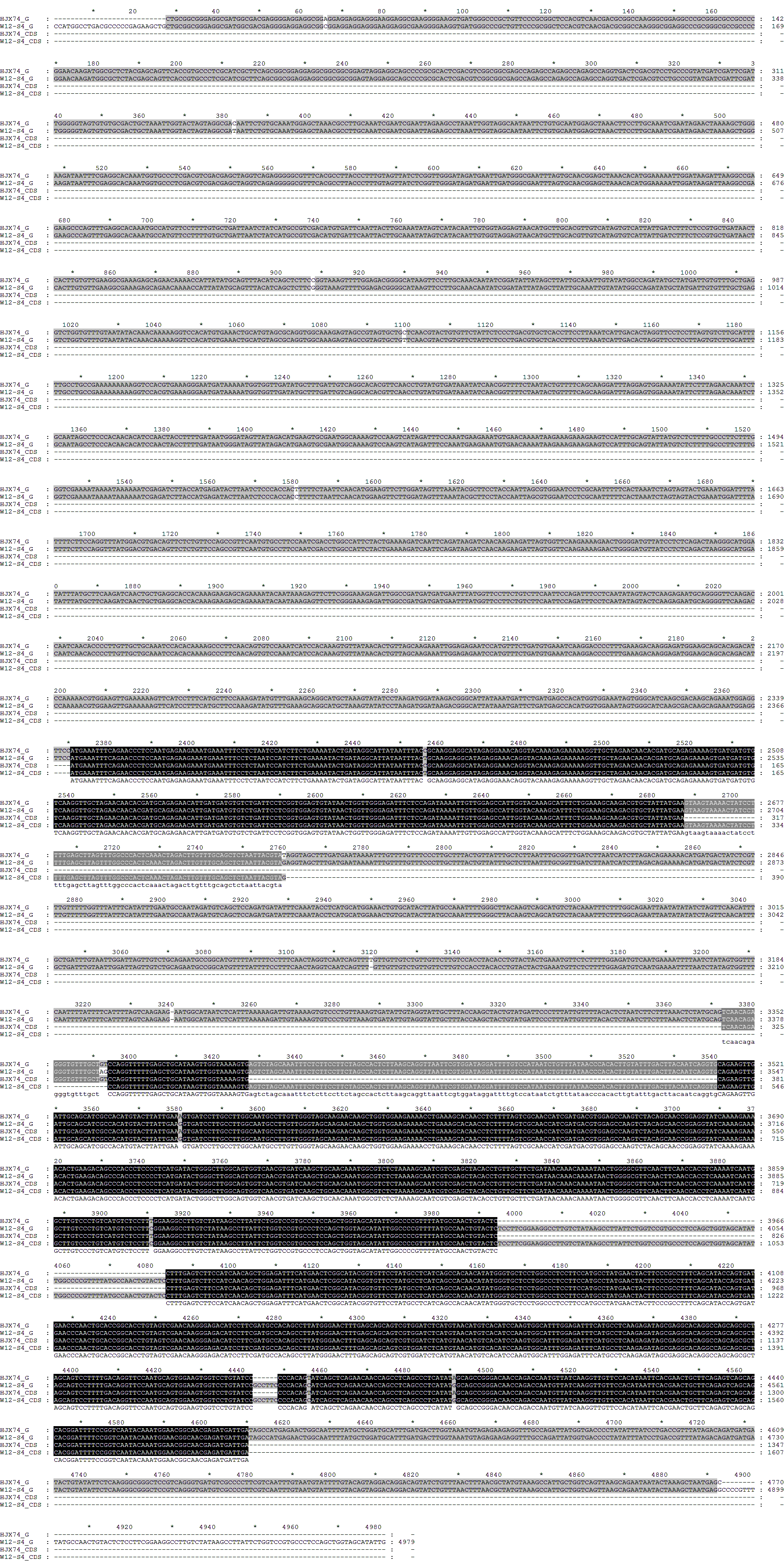
S6 Fig. Comparative analysis of genomic DNA and cDNA of *Hd17* gene.

Supplement: S6 Fig — (DOC) [file pone.0190491.s009.doc]

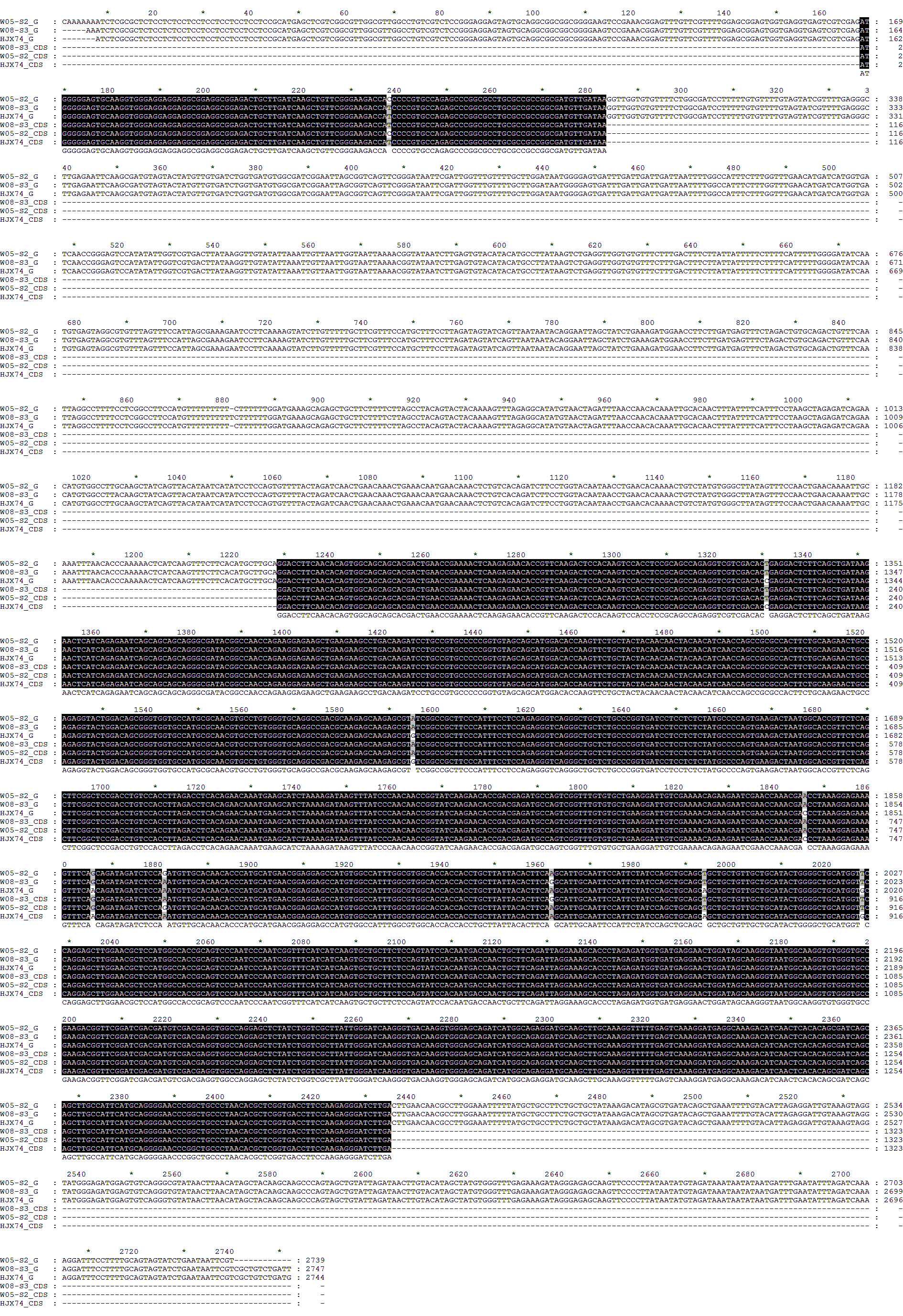


S7 Fig. Comparative analysis of genomic DNA and cDNA of *OsDof12* gene.

Supplement: S7 Fig — (DOC) [file pone.0190491.s010.doc]

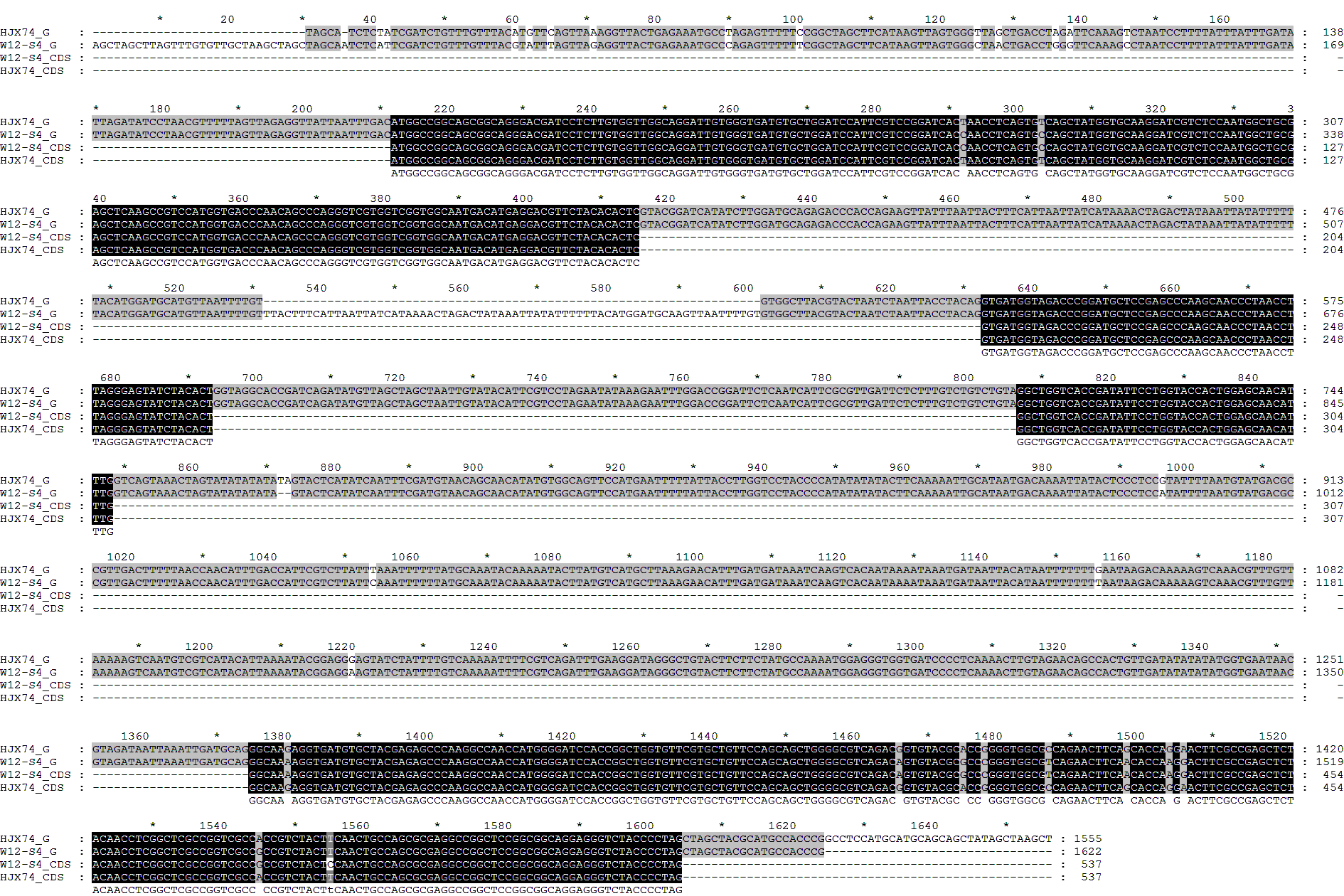


S8 Fig. Comparative analysis of genomic DNA and cDNA of *RFT1* gene.

Supplement: S8 Fig — (DOC) [file pone.0190491.s011.doc]
